# Thalamus-driven functional populations in frontal cortex support decision-making

**DOI:** 10.1038/s41593-022-01171-w

**Published:** 2022-09-28

**Authors:** Weiguo Yang, Sri Laasya Tipparaju, Guang Chen, Nuo Li

**Affiliations:** Department of Neuroscience, Baylor College of Medicine, Houston, TX USA

**Keywords:** Decision, Neural circuits, Neural encoding

## Abstract

Neurons in frontal cortex exhibit diverse selectivity representing sensory, motor and cognitive variables during decision-making. The neural circuit basis for this complex selectivity remains unclear. We examined activity mediating a tactile decision in mouse anterior lateral motor cortex in relation to the underlying circuits. Contrary to the notion of randomly mixed selectivity, an analysis of 20,000 neurons revealed organized activity coding behavior. Individual neurons exhibited prototypical response profiles that were repeatable across mice. Stimulus, choice and action were coded nonrandomly by distinct neuronal populations that could be delineated by their response profiles. We related distinct selectivity to long-range inputs from somatosensory cortex, contralateral anterior lateral motor cortex and thalamus. Each input connects to all functional populations but with differing strength. Task selectivity was more strongly dependent on thalamic inputs than cortico-cortical inputs. Our results suggest that the thalamus drives subnetworks within frontal cortex coding distinct features of decision-making.

## Abstract (web summary)

Frontal cortex contains a complex mixture of signals reflecting distinct behavioral and cognitive processes. An analysis of 20,000 neurons during decision-making revealed distinct functional clusters and their activities are driven by the thalamus.

## Main

During perceptual decision-making, frontal cortical neurons exhibit diverse selectivity representing sensory, motor and cognitive variables^1–3^. The circuit underpinning this diverse selectivity remains poorly understood. One view posits that a shared neuronal population multiplexes multiple computations^4–8^. This view is supported by neurophysiology recordings that show individual neurons exhibit a seeming continuum of time-varying responses and random combinations of task selectivity^1,7,9,10^. Randomly mixed selectivity produces high-dimensional representations and greater computational capacity^10,11^. Mixed selectivity could arise in recurrent neural networks with little circuit structure^1,6,11^. In this scheme, single neuron responses cannot be readily interpreted in terms of anatomical circuit organization. On the other hand, anatomically defined neurons in frontal cortex are found to encode specific aspects of behavior^12–16^. Cell-type-specific coding implies a structured underlying circuit, whereby segregated populations carry out specific computations. A class of recurrent network models rely on segregated functional populations coding specific features of behavior^17,18^. It remains poorly understood if and how neural coding of behavioral information is related to the anatomical organization of frontal cortical circuits.

We set out to address two related questions: (1) how information supporting a perceptual decision is encoded by frontal cortical neurons; (2) how the encoding is related to the anatomical circuit organization. Frontal cortical circuits have highly organized anatomical structure^19,20^. For example, in mouse vibrissal motor cortex, inputs from somatosensory cortex preferentially innervate superficial layers whereas thalamic inputs target deep layer neurons^20,21^. Superficial layer neurons preferentially project back to somatosensory cortex and deep layer neurons project back to the thalamus^21^, forming distinct long-range loops^22^. Frontal cortex also forms reciprocal loops with the thalamus and other cortical regions to maintain persistent activity^23–25^. Previous studies found distinct frontal cortex projection neurons carrying specific information to different brain regions^12–16^. However, no study has related neural coding in frontal cortex to long-range input connectivity. A key question is how inputs from different brain regions produce the complex selectivity in frontal cortex.

The mouse anterior lateral motor cortex (ALM) is necessary for perceptual decisions^24,26–31^. We analyzed activity of 20,000 ALM neurons during tactile decision-making. Individual neurons conformed to a collection of prototypical response profiles that were repeatable across mice. Contrary to the notion of randomly mixed selectivity, activity signaling stimulus, choice and action were supported by distinct but partially overlapping functional populations that could be delineated by their response profiles. We related the functional populations to long-range inputs from somatosensory cortex, contralateral hemisphere (contralateral ALM (cALM)) and thalamus. Each input targeted all functional populations and contributed to task selectivity, but with differing strengths. Task selectivity was more strongly dependent on thalamic inputs than cortico-cortical transmission. Our results suggest that the thalamus drives subnetworks within frontal cortex coding specific features of perceptual decision-making.

## Results

### Analysis of 20,000 neurons reveals repeatable response profiles in ALM

**Fig. 1:**
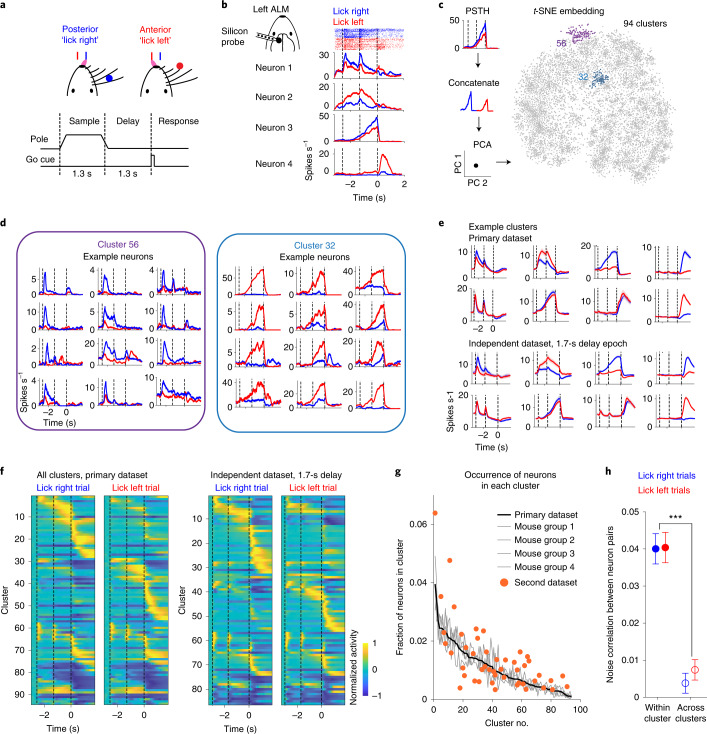
Diverse yet repeatable response profiles in ALM. **a**, Mice reporting the location of a pole by directional licking after a delay epoch. **b**, Silicon probe recording and example neurons. Left ALM. Top: spike raster. Bottom: PSTH. Blue, ‘lick right’ trials; red, ‘lick left’. Dashed lines, behavioral epochs as in **a**. **c**, Analysis of diverse response profiles. Individual neuron PSTHs of ‘lick right’ (blue) and ‘lick red’ trials (red) are concatenated. The population response is reduced to the top 50 principal components and embedded into a two-dimensional *t*-SNE. Dots, individual neurons. Only neurons showing consistent modulation during the task are included (*n* = 7,340 neurons, 73 mice). Neurons are divided into 94 clusters. Colors show two clusters. PC, principal component. **d**, PSTHs of individual neurons in the example clusters in **c**. **e**, Rows 1–2: PSTHs (mean ± s.e.m. across neurons) of eight example clusters in the primary dataset. Rows 3–4: PSTHs of matching clusters from a second dataset (*n* = 8,736 neurons, 29 mice). *t*-SNE and clustering are performed independently on the second dataset, resulting in 86 clusters. **f**, Left: response profiles of all clusters from the primary dataset. Each row shows activity of one cluster. Right: response profiles of the second dataset. **g**, Fraction of neurons falling into each cluster in the primary dataset (thick line). Clusters are ranked based on size. Gray lines, fraction of neurons in four distinct mouse groups (18 mice each; *n* = 1,628, 2,095, 1,547, 1,984 neurons, respectively). Dots, fraction of neurons in matched clusters from the second dataset. The position of the dots on the *x* axis is based on the matching cluster from the primary dataset. **h**, Noise correlation for simultaneously recorded neuron pairs. *n* = 1,060 pairs from the same cluster (filled symbols); *n* = 1,598 pairs from different clusters (open symbols) (Methods). Noise correlation is trial-to-trial cofluctuations in the mean-subtracted spike rate. See Extended Data Fig. 1i for noise correlation during specific task epochs. Mean ± s.e.m. across neuron pairs. ***P = 1.82 × 10^−49^, two-sided Wilcoxon rank sum test, within-cluster pairs versus across-cluster pairs. ‘Lick right’ and ‘lick left’ trials are combined for the test.

Do frontal cortical neurons exhibit a continuum of responses during perceptual decision-making or conform to a fixed set of prototypical response profiles? A continuum of responses could imply little underlying circuit structure^1,6^. To answer this question, we recorded activity from ALM neurons during a tactile decision task (Fig. 1a and Methods). Mice discriminated the location of an object (anterior or posterior) using their whiskers and reported choice using directional licking (‘lick left’ or ‘lick right’) to obtain a water reward. A delay epoch separated the sensory stimulus and behavioral response, and an auditory ‘go’ cue signaled the onset of response. We used silicon probes to record 23–43 neurons at a time (Methods, all recordings were from left ALM). Across numerous recordings, we obtained responses from 9,626 ALM neurons (347 sessions, 73 mice). In most experiments, the delay epoch was 1.3 s. In parallel, we analyzed an independent dataset in which mice performed the same task with a longer delay (1.7 s; 10,420 neurons, 110 sessions, 29 mice; datasets from ref. ^24^ and ref. ^31^).

**Extended Data Fig. 1:**
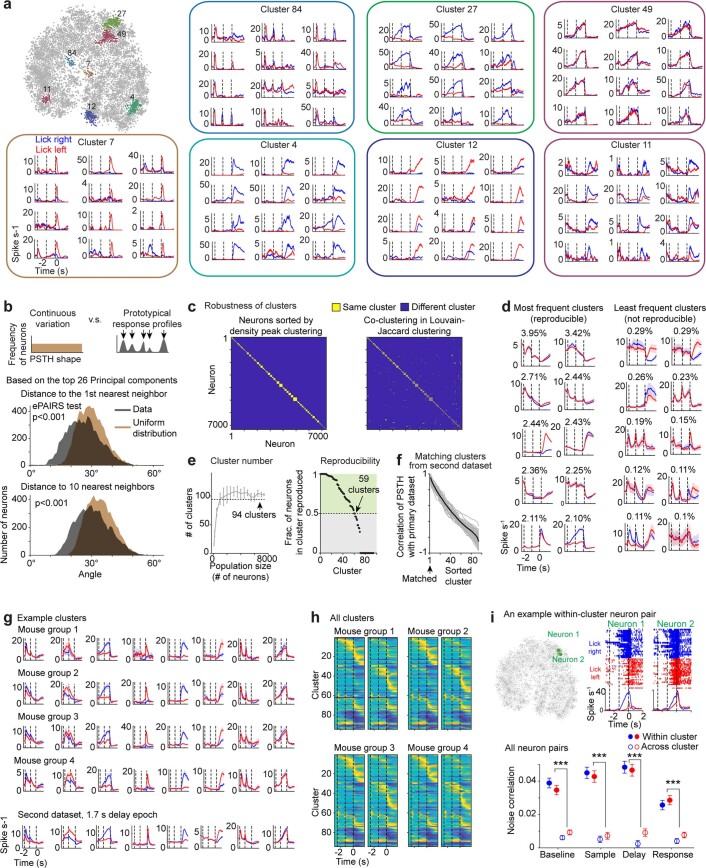
Prototypical response profiles in ALM. **a** Example clusters from the primary dataset. *Left*, dots represent individual neurons in the t-SNE representation. Neurons are divided into 94 clusters. Colors indicate 7 example clusters. *Right*, PSTHs of individual neurons in the example clusters. **b** ePAIRS test for distribution of response profiles. Each neuron’s PSTH shape is represented by a 26-dimensional vector that contains the loadings of the top 26 principal components of the population activity. For continuous variation of response profiles (that is no clustering), the vectors are uniformly distributed around the origin, which can be quantified by computing the angle between nearest neighbors (ePAIRS test). The distribution of angles deviates significantly from uniform distribution (*P* <1 × 10^−4^ for k=1, *P* < 1 × 10^−4^ for k=10, one-sided test, Methods), indicating that ALM response profiles exhibit clusters of prototypical response profiles. The result is consistent across different number of nearest neighbors (*k*) used to calculate average angle. **c** Cell-cell co-clustering matrix for every pair of neurons in the primary dataset. Only neurons showing consistent modulation during the task are included (n = 7340 neurons, 73 mice). Neurons are sorted based on density peak clustering (*left*). Co-clustering matrix of the same neuron pairs is shown for Louvain-Jaccard clustering (*right*). The block structure along the matrix diagonal is preserved in Louvain-Jaccard clustering, indicating that if two neurons belong to the same cluster by density peak clustering, then their co-clustering probability is high for Louvain-Jaccard clustering. **d** Average PSTHs of the largest and smallest clusters from the primary dataset. Mean ± SEM across neurons. The largest clusters are reproducible in Louvain-Jaccard clustering. The small clusters are not reproduced. **e** Robustness of the clustering results. *Left*, number of clusters from density peak clustering as a function of population size. Neurons are subsampled. Mean ± S.D. across populations. Cluster number saturates rapidly after 1000 neurons. Dashed line, the primary dataset consists of 94 clusters after manual merging of some similar clusters (Methods). *Right*, reproducibility of clusters in Louvain-Jaccard clustering. For each cluster from density peak clustering, we quantified the fraction of its units captured by a matching cluster in Louvain-Jaccard clustering (Methods). Clusters with >0.5 of units captured are considered reproducible. **f** Clusters from the second dataset are matched to clusters from the primary dataset based on the similarity their PSTHs. The plot shows Pearson’s correlation between clusters from the second dataset with all the clusters from the primary dataset. Clusters are matched based on high correlation coefficient (Methods). This analysis focuses on 48 clusters from the second dataset that are most readily matched to a corresponding cluster in the primary dataset. Gray lines, individual clusters; black line, mean. **g** Average PSTHs of 8 example clusters. Mean ± SEM across neurons. Rows 1–4 show distinct mouse groups (n = 18 mice per group). Row 5 shows matching clusters from the second dataset. **h** Response profiles of all clusters from 4 distinct mouse groups. Each row shows average activity of one cluster. **i** Neuron pairs with similar response profiles exhibit noise correlation. *Top*, an example neuron pair from the same cluster. Spike raster and PSTHs show simultaneously recorded responses from the neuron pair. In trials where one neuron exhibits high spike rate, the other neuron also exhibits high spike rate. The two neurons are 200 µm apart. *Bottom*, noise correlation for all neuron pairs. Mean ± SEM across neuron pairs. Same as Fig. 1h but for noise correlation calculated in various epochs. Baseline, = 1.98 × 10^−11^; sample, *P* = 1.94 × 10^−30^; delay, *P* = 8.91 × 10^−35^; response, *P* = 9.92 × 10^−15^, two-sided Wilcoxon rank sum test (Methods).

Individual ALM neurons exhibited diverse response profiles^1,7,9,14^, including activity during the tactile stimulus, persistent or ramping activity during the delay epoch, and activity during the response epoch (Fig. 1b). However, we observed that many neurons frequently exhibited the same peri-stimulus time histograms (PSTHs) (Fig. 1c,d and Extended Data Fig. 1a). To examine the distribution of ALM responses, we assembled the PSTHs into a population response matrix (*neurons* × *time steps*, ‘lick right’ and ‘lick left’ trials concatenated, correct trials only). We performed principal component analysis (PCA) on the response matrix and characterized individual neuron PSTH shapes as 26-dimensional vectors using the top 26 principal components (capturing 98% of activity variance). We then used a nonparametric statistical test to examine if the 26-dimensional vectors were uniformly distributed, that is, a continuum of response profiles^9,12^ (elliptical projection angle index of response similarity (ePAIRS) test, Methods). The distribution was highly nonuniform (*P* < 0.001; Extended Data Fig. 1b), which indicated that groups of ALM neurons exhibited similar response profiles (Fig. 1c,d and Extended Data Fig. 1a).

To visualize the repertoire of response profiles, we embedded the activity of ALM neurons into a two-dimensional representation based on the similarity of PSTHs (Fig. 1c and Extended Data Fig. 1a; *t*-distributed stochastic neighbor embedding (*t*-SNE)). We divided the neurons into 94 putative clusters that corresponded to distinct response profiles (Fig. 1e,f, Extended Data Fig. 1c–e and Methods). Examination of individual clusters confirmed that the same PSTH was frequently repeated in individual neurons (Fig. 1d and Extended Data Fig. 1a). The majority of the clusters (59 of 94 clusters, containing 74.2% of neurons) were reproducible across clustering methods, while the smaller clusters were not always recovered (Extended Data Fig. 1c–e). Robust clusters thus defined a set of prototypical response profiles in ALM. Defined clusters provided a way to compare response profiles across datasets by examining matched clusters (Extended Data Fig. 1f).

The prototypical response profiles were highly repeatable across mice. We divided the dataset into four subsets with different groups of mice (Extended Data Fig. 1g,h). We treated groups of mice because a large number of neurons was needed to sufficiently cover the full collection of response profiles (Extended Data Fig. 1e). The fraction of neurons exhibiting each prototypical response profile was consistent across mouse groups (Fig. 1g), indicating that the response profiles in different mice followed a consistent distribution. Remarkably, the same collection of response profiles was also observed in the second dataset (Fig. 1e,f and Extended Data Fig. 1g, 1.7-s delay), even though *t*-SNE and clustering were performed independently. Importantly, for matched clusters, the fraction of neurons exhibiting each prototypical response profile was also consistent across datasets (Fig. 1g). Notably, neurons with similar response profiles exhibited significant trial-to-trial correlation in activity compared with neurons with distinct response profiles (Fig. 1h). Activity was correlated even before the trial started (Extended Data Fig. 1i). This suggests that neurons with similar response profiles belong to subnetworks.

Thus, ALM neurons exhibited a repeatable collection of prototypical responses. The repeatable response profiles made circuit analysis possible. Sampling neurons from different mice could recover the same collection of responses. This permitted us to examine how behavioral information was encoded by defined neuronal populations and relate the neural coding to anatomical circuit organization.

### Stimulus, choice and action are encoded by distinct activity modes

We first examined how task and behavioral information was encoded by ALM population activity. We considered four trial types: correct trials in which mice licked as instructed by object location (anterior, lick left; posterior, lick right) and were rewarded; and error trials in which mice licked the other lickport (anterior, lick right; posterior, lick left) and were not rewarded. Trial types thus differed in object location (‘stimulus’, anterior versus posterior), lick direction (‘choice’, left versus right) and reward (‘outcome’, rewarded versus unrewarded).

**Fig. 2:**
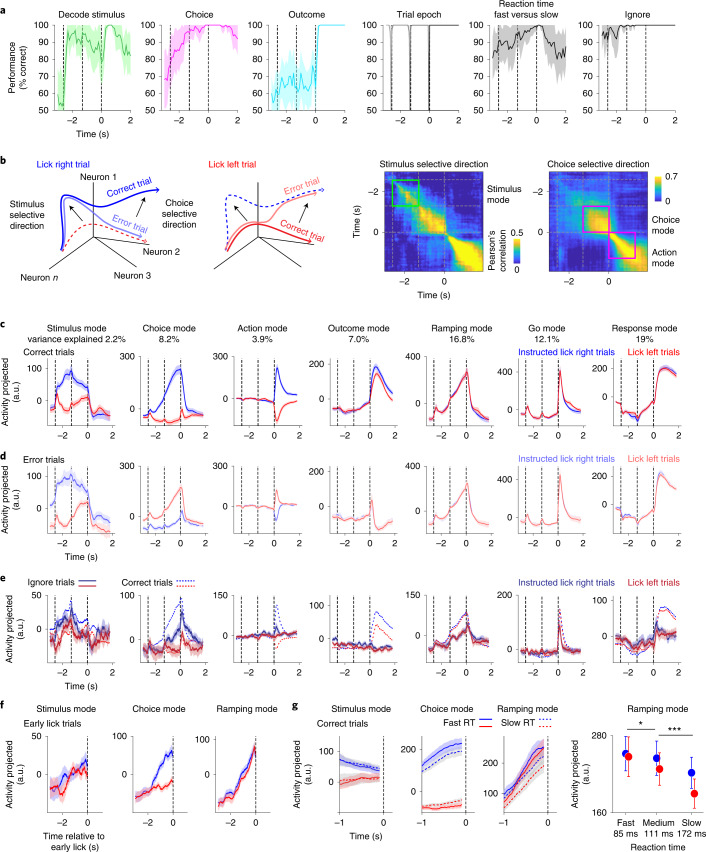
Task and behavioral information is represented by distinct activity modes. **a**, Decoding accuracy for stimulus (trial type instructed by object location), choice (lick direction), outcome (rewarded versus unrewarded), trial epoch (baseline, sample, delay, response), reaction time (fast versus slow trials) and ignore trials. Decoding is performed independently at each time point on population responses generated from different combinations of single neuron trial data (mean ± s.d. across runs, Methods). Only neurons with more than ten error trials of each trial type are included (*n* = 2,039). **b**, Left: neural trajectories and selectivity directions in activity space. Error trial trajectories distinguish stimulus versus choice selectivity. Right: correlation of stimulus and choice selective directions across time. Bounding boxes, activity modes are selective directions in specific epochs. Green, stimulus mode. Magenta, choice and action modes. Only neurons with more than five error trials of each trial type are included (*n* = 3,966). **c**, ALM population activity along specific activity modes in correct trials. Mean ± s.e.m. (bootstrap, Methods). Blue, ‘lick right’ trials instructed by object location; red, ‘lick left’ trials. Percentage of activity variance captured is shown at top. **d**, Activity projection in error trials. Mean ± s.e.m. (bootstrap, Methods). Light blue, ‘lick right’ trials instructed by object location, but mice licked left; light red, ‘lick left’ trials in which mice licked right. **e**, Activity projection in ignore trials. Mean ± s.e.m. (bootstrap, Methods). Activity modes are computed separately from **c** and **d** using neurons with more than two ignore trials of each trial type (*n* = 546). Dashed lines, activity in correct trials. Dark blue, ‘lick right’ trials instructed by object location; dark red, ‘lick left’ trials. **f**, Activity projection in trials in which mice licked before the go cue. Activity is aligned to the first lick. Mean ± s.e.m. (bootstrap, Methods). Only neurons with more than three early lick trials of each trial type are included (*n* = 1,994). Also see Extended Data Fig. 3. **g**, Left: activity projection separately by fast or slow reaction time. Top and bottom 1/3 of trials sorted by reaction time. Correct trials only. The *x* axis is the same as in panels **c** and **d**. Mean ± s.e.m. (bootstrap, Methods). Right: activity projection during the last 200 ms of the delay epoch. Trials with the fastest (top 1/3), intermediate (middle 1/3) and slowest reaction times (bottom 1/3). Mean ± s.e.m. (bootstrap, Methods). Only neurons with more than five error trials and more than two trials of each reaction time condition are included (*n* = 3,918). *P = 0.002; ***P = 5.06 × 10^−14^, two-tailed *t*-test. a.u., arbitrary units; RT, reaction time.

Information about stimulus, choice and outcome was readily available for ALM population activity^32^. We trained linear decoders on single-trial population activity to differentiate stimulus, choice or outcome (Methods). Stimulus information increased rapidly at the onset of the sample epoch and persisted through the delay epoch (Fig. 2a). The persistent stimulus information might represent a memory of object location. Choice information increased gradually during the sample epoch and reached the maximum just before the response epoch (Fig. 2a). Outcome information was mostly available during the response epoch (Fig. 2a). In addition, the trial temporal structure (that is, epoch identity) could be read out from ALM activity (Fig. 2a). In correct trials, reaction time of the first lick could be predicted from activity well before the motor response (Fig. 2a, fast versus slow reaction time; 85 ± 24 and 172 ± 103 ms, respectively, mean ± s.e.m.). Activity also predicted trials in which mice did not lick after the ‘go’ cue (ignore trials, Fig. 2a). Because ALM activity is strongly influenced by ongoing movements^33,34^, a portion of the decoded information may also reflect differences in uninstructed movements between trial types.

**Extended Data Fig. 2:**
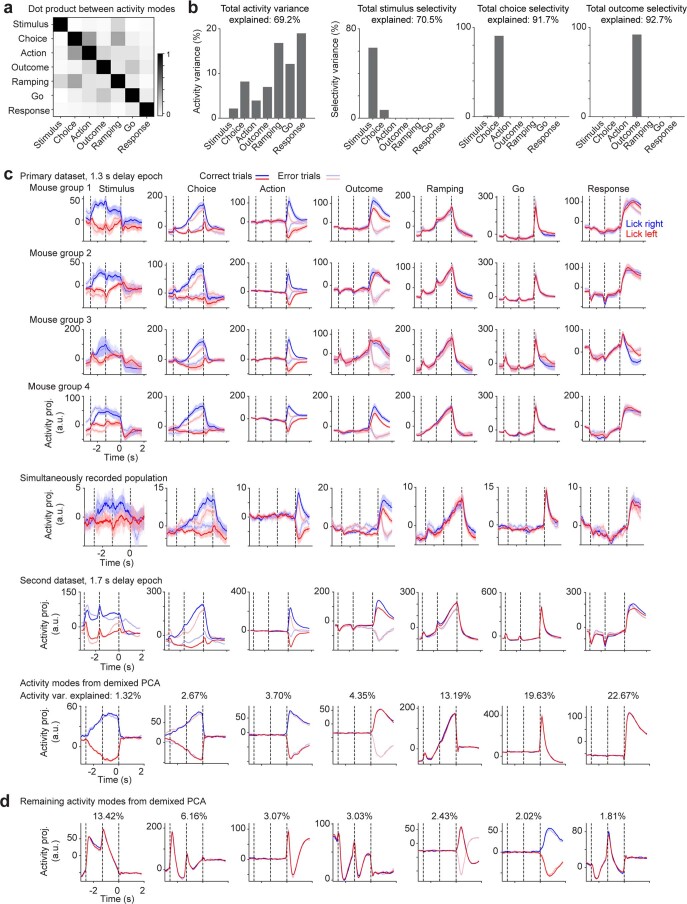
Activity modes are highly robust across mice, conditions, and analysis Methods. **a** Angles between activity modes. The activity modes are near orthogonal to each other. **b** Activity and selectivity variance explained by each activity mode. Activity and selectivity variance are computed using trial-averaged activity during specific epochs (Methods), thus they reflect variance across time and neurons. Variance across trials is not reflected. **c** Activity modes are highly robust across mice, conditions, and decomposition methods. The plots show ALM activity projections on specific activity modes. Solid colors, correct trials; transparent colors, error trials. Blue, ‘lick right’ trials instructed by object location; red, ‘lick left’ trials. Row 1–4, activity modes from 4 distinct mouse groups in the primary dataset. Only neurons with more than 5 error trials of each trial type are included (n = 988, 767, 972, 1200 respectively). Mean ± SEM (bootstrap, Methods). Row 5, activity modes from simultaneously recorded populations (33 sessions, 10–57 neurons per session, average 24 neurons). Mean ± SEM across sessions. Row 6, activity modes from the second dataset (n = 4010 neurons). Mean ± SEM (bootstrap, Methods). Row 7, activity modes from demixed principal component analysis (demixed PCA) on the primary dataset. The top activity modes discovered by demixed PCA correspond to stimulus, choice, action, outcome, ramping, go, and response mode. The percentage of activity variance captured by each activity mode is shown on top. **d** Additional activity modes obtained from demixed PCA ranked by their activity variance. Together, the 14 activity modes from demixed PCA shown here captured 99.47% of activity variance.

To understand how information was encoded by ALM populations, we analyzed ALM activity in an activity space where individual dimensions corresponded to the activity of individual neurons. We decomposed ALM activity into several activity modes, corresponding to distinct directions in activity space along which activity was selective for stimulus, choice or action (Fig. 2b and Methods)^8,24,35^. We calculated the selective directions at different times of the trial (Fig. 2b). Stimulus information before the motor response was strongest during the sample epoch (Fig. 2a) and stimulus selective direction was similar during this epoch (Fig. 2b, green bounding box), and we therefore defined this direction as the stimulus mode. Its activity projection exhibited persistent trial-type information during both the sample and delay epochs (Fig. 2c). Based on the choice selective direction during the delay epoch, we determined a choice mode (Fig. 2b). This activity projection exhibited ramping selectivity for upcoming lick direction (Fig. 2c). The choice mode collapsed during the response epoch and a new choice selective direction developed (Fig. 2b)^24,35,36^. ALM activity is necessary for licking response^14,35,37^. We therefore defined the choice selective direction in the response epoch as an action mode (Fig. 2c). Finally, we determined an outcome mode that differentiated rewarded and unrewarded trials during the response epoch (Fig. 2c). The activity modes were near orthogonal to each other (Extended Data Fig. 2a). Thus, ALM signaled stimulus, choice and action along near orthogonal directions in activity space.

We additionally determined three non-trial-type-selective activity modes based on previous studies (Methods). One activity mode captured nonselective ramping activity during the delay epoch (ramping mode, Fig. 2c), which might reflect an urgency signal or passage of time^24,30,38–40^. Another activity mode with phasic activity after the ‘go’ cue was important for triggering the motor response (go mode, Fig. 2c)^35^. We defined ramping and cue modes as in refs. ^30,35^, which by construction captured activity showing a ramp during the delay, and a phasic response after the go cue. Finally, the activity mode explaining the most variance showed nonselective modulation during the motor response (response mode, Fig. 2c), consistent with previous decompositions of frontal cortex dynamics^7,41^. Together, the seven activity modes captured 69% of variance in ALM population activity (Extended Data Fig. 2b), including most of the stimulus and choice selectivity (71% and 92%; Extended Data Fig. 2b).

**Extended Data Fig. 3:**
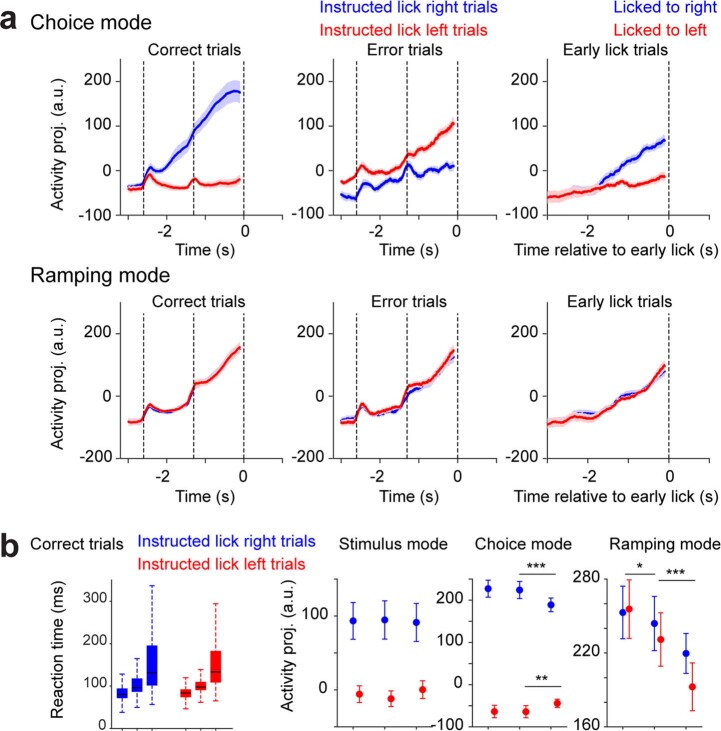
Correlation of activity modes with behavior. **a** Activity projections in correct, error, and early lick trials. Activity is aligned to the go cue for correct and error trials. Activity is aligned to the time of the first lick for early lick trials. Only neurons with more than 3 early lick trials of each trial type are included (n = 1994). Mean ± SEM (bootstrap, Methods). **b** *Left*, reaction time in correct trials with the fastest (top 1/3), intermediate (middle 1/3), and slowest reaction time (bottom 1/3). Box-whisker plot, box edges, 75 and 25 percentiles, whiskers, extremities of data not considered as outliers, black bars, median. n = 132,907 trials/85 mice. *Right*, activity projections during the last 200 ms of the delay epoch. Only neurons with more than 5 error trials of each trial type and more than 2 trials of each reaction time condition are included (n = 3918). Choice mode, **P = 0.0014; ***P = 3.22 × 10^−5^, two-tailed t-test. Ramping mode, *P = 0.002; ***P = 5.06 × 10^−14^, two-tailed t-test.

Importantly, distinct activity modes predicted different features of behavior. In error trials, the stimulus mode signaled trial type irrespective of lick direction whereas the choice and action modes tracked mice’s lick directions (Fig. 2d). The choice and ramping modes also correlated with ignore and early lick behaviors even though the activity modes were defined without considering these conditions. In ignore trials, activity increased less along the choice and ramping modes (Fig. 2e). When mice licked before the ‘go’ cue, ramping activity preceded licking and the choice mode predicted lick direction (Fig. 2f and Extended Data Fig. 3a). This suggests that the choice and ramping modes were related to upcoming licks. Further supporting this interpretation, the choice and ramping modes predicted mice’s reaction times in correct trials (Fig. 2g). In contrast, activity along the stimulus mode did not predict early lick or reaction time (Fig. 2f,g and Extended Data Fig. 3b).

The same set of activity modes were reliably obtained in different mice and datasets (Extended Data Fig. 2c). Although our analyses used neurons combined from different recordings, decomposition of simultaneously recorded populations obtained the same activity modes (Extended Data Fig. 2c). Importantly, a different dimensionality reduction method, demixed PCA^7^, also discovered the same set of activity modes (Extended Data Fig. 2c,d). These analyses suggest that the activity modes captured prominent components of ALM activity encoding specific behavioral features.

### Stimulus, choice and action are coded by largely segregated neuronal populations

Do shared neuronal populations support different activity modes? Recordings in frontal and parietal cortex previously found that selectivity for stimulus, choice and action is randomly mixed across shared neuronal populations^1,7,9^. Randomly mixed neural coding implies a shared network multiplexes multiple computations. Taking advantage of the large number of neurons in our dataset and the repeatable response profiles, we examined how distinct activity modes were distributed across defined ALM populations.

**Fig. 3:**
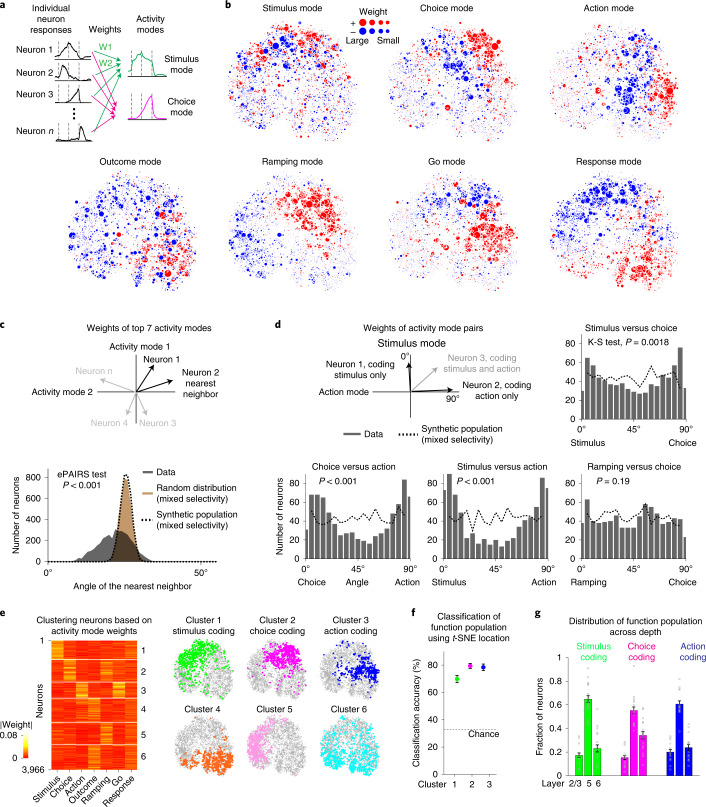
Activity modes coding stimulus, choice and action are supported by distinct neuronal populations. **a**, Activity modes correspond to weighted sums of individual neuron activities. The weights show contribution of individual neurons. **b**, Neuron weights in the *t*-SNE. Dots, individual neurons. Dot size shows weight magnitude and colors indicate positive (red) or negative (blue) weights. Only neurons with more than five error trials of each trial type are included (*n* = 3,966). **c**, Top: a seven-dimensional vector represents each neuron’s contributions to the activity modes. For neuronal populations with random mixtures of selectivity, coding vectors are uniformly distributed around the origin, which can be quantified by angles between nearest neighbors (ePAIRS test). Bottom: the distribution of angles deviates significantly from random distribution of coding vectors and from a synthetic population coding random mixtures of activity modes, indicating that distinct task selectivity is not randomly mixed within ALM populations. *P* < 1 × 10^−4^, one-sided test (Methods). **d**, A two-dimensional vector represents each neuron’s contributions to a pair of activity modes. If neurons encode random mixtures of each activity mode, rather than encoding one mode or another, these vectors are uniformly distributed. Neuronal populations coding single activity modes are located around 0° and 90°. Neural coding of stimulus, choice and action exhibits significant peaks at 0° and 90°. In contrast, coding of choice and ramping shares the same neuronal population. Dashed line, synthetic population coding mixtures of activity modes. Stimulus and choice, *P* = 0.0018; choice and action, *P* = 4.40 × 10^−6^; stimulus and action, *P* = 2.27 × 10^−9^; ramping and choice, *P* = 0.19, Kolmogorov–Smirnov test, observed distribution versus synthetic population, one-sided test. **e**, Left: *k*-means clustering on activity mode weights delineates neurons into six clusters (Methods). Right: clusters shown in the *t*-SNE. Clusters carrying the most variance for the stimulus, choice and action modes are termed stimulus, choice and action coding (Extended Data Fig. 5a). **f**, Classification of stimulus, choice and action coding neurons using a nearest-neighbor classifier in the *t*-SNE (Methods). Mean ± s.e.m. (bootstrap across neurons). Only neurons with more than five error trials of each trial type are included (*n* = 3,966). **g**, Distribution of stimulus, choice and action coding neurons across depth. Fraction is relative to all neurons from each functional population (stimulus coding, *n* = 583 neurons/73 mice; choice coding, *n* = 694 neurons/73 mice; action coding, *n* = 491 neurons/73 mice). Mean ± s.e.m. across mice (dots). K-S, Kolmogorov–Smirnov test; W, weight.

**Extended Data Fig. 4:**
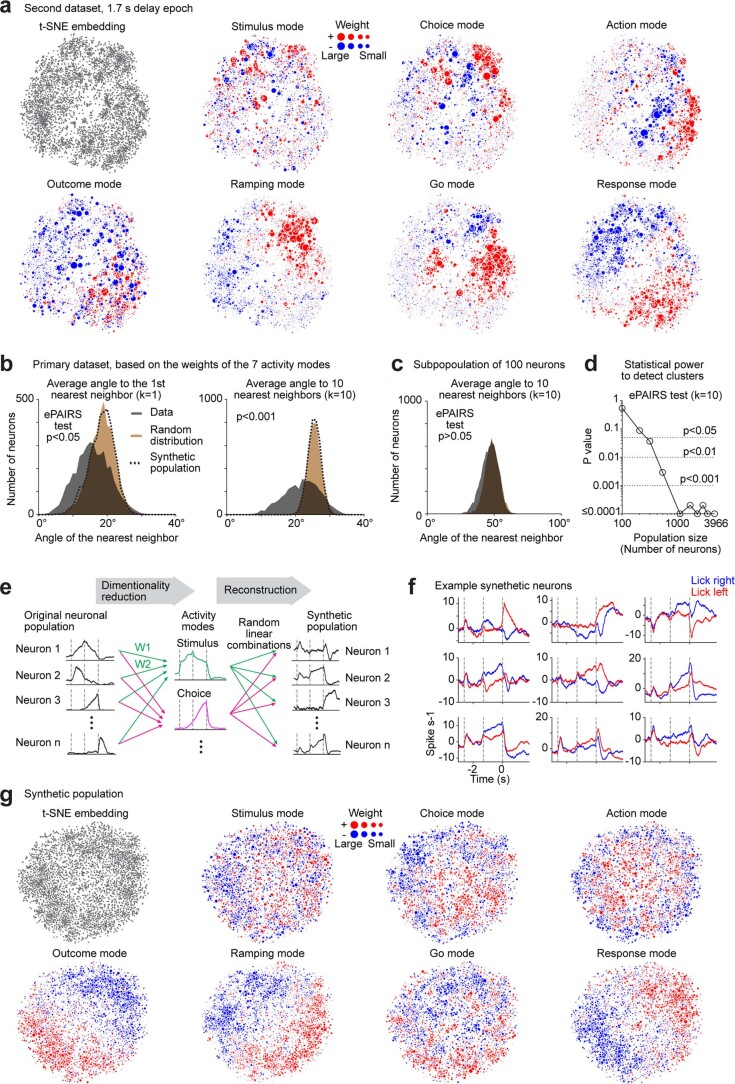
Activity modes are non-randomly mixed across ALM populations. **a** Neuron weights in the t-SNE representation. Same as Fig. 3b, but for the second dataset. **b** Distribution of coding vector angles between nearest neighbors. Same as Fig. 3c, but for different number of nearest neighbors (*k*) used to calculate average angles (ePAIRS test, one-sided test; *P* = 0.049 for k=1, *P* < 1 × 10^−4^ for k=10, Methods). Data from the primary dataset. Only neurons with more than 5 error trials of each trial type are included (n = 3966). **c** Effect of neuronal population size on analysis of mixed selectivity. 100 neurons are randomly selected from the full dataset to generate subpopulations. The histogram shows the distribution of coding vector angles between nearest neighbors in the subpopulation (*P* = 0.54, ePAIRS test, one-sided test). The distribution is not significantly different from random distribution. Thus a population of 100 neurons appears to exhibit randomly mixed selectivity. **d** Power analysis showing the P value of ePAIRS test as a function of population size. Subsets of neurons are randomly selected from the full dataset to generate subpopulations. Detecting significant deviations from randomly mixed selectivity using a criterion of *P* < 0.01 (one-sided test) requires at least 400 neurons. **e** Generation of a synthetic population in which the coding of activity modes is randomly mixed. Each synthetic neuron’s PSTH is constructed from random combinations of the activity modes and eigenvectors of the original population response. This procedure preserved the activity modes but redistributed them randomly across the synthetic population. **f** PSTHs of example synthetic neurons. Blue, ‘lick right’ trials; red, ‘lick left’ trials. **g** Neuron weights in the t-SNE representation. Same as Fig. 3b, but for the synthetic population.

Each activity mode is a weighted sum of individual neuron activities. The weights show the contribution of individual neurons (Fig. 3a). We visualized the neuron weights for each activity mode in the *t*-SNE representation. Neurons supporting stimulus, choice and action modes were clustered to different locations in the *t*-SNE (Fig. 3b), suggesting that stimulus, choice and action activity may be signaled by neuronal populations with different response profiles. Interestingly, neurons supporting the ramping mode co-localized to the same location in the *t*-SNE as the choice coding neurons (Fig. 3b), suggesting that a shared neuronal population signals choice and ramping activity. The same pattern of weight distribution was reproduced in the second dataset (Extended Data Fig. 4a).

We took two different approaches to quantitatively test whether activity modes were supported by shared or distinct neuronal populations. First, we examined individual neuron weights for all seven activity modes. Each neuron is thus characterized by a seven-dimensional coding vector. If activity modes are randomly mixed across ALM populations, coding vectors are uniformly distributed around the origin (Fig. 3c and Extended Data Fig. 4b)^7,9^. Instead, ALM coding vectors were highly nonrandom and exhibited significant clustering (Fig. 3c and Extended Data Fig. 4b; *P* < 0.001 ePAIRS test, Methods)^12^. To examine how specific activity modes were supported by ALM populations, we examined neuron weights for pairs of activity modes: stimulus versus choice, choice versus action or stimulus versus action (Fig. 3d). The angles of the two-dimensional coding vectors were clustered near 0° and 90°, which correspond to separate populations coding one of the activity modes but not the other (Fig. 3d). A portion of the coding vectors were near 45°, which corresponds to neurons carrying mixtures of selectivity. However, the binomial distribution indicates that ALM neurons encoding one task variable were less likely to contribute to another. In contrast, the coding vectors for choice and ramping modes were uniformly distributed (Fig. 3d). This confirms that the choice and ramping modes were coded by shared neuronal populations (Fig. 3b).

To contrast with the actual ALM population, we generated a synthetic neuronal population which encoded random mixtures of activity modes by construction. We synthesized individual neuron responses from random linear combinations of the activity modes (Extended Data Fig. 4e and Methods). Thus, the activity modes were preserved at the level of the population, but the contribution of individual neurons was scrambled. The synthetic neurons exhibited heterogeneous responses similar to the actual ALM neurons (Extended Data Fig. 4f). We performed *t*-SNE of the synthetic responses. In contrast to the data, activity modes were more scattered across the *t*-SNE (Extended Data Fig. 4g). Individual neuron coding vectors were uniformly distributed around the origin, deviating significantly from the data (Fig. 3c,d and Extended Data Fig. 4b; *P* < 0.001).

**Extended Data Fig. 5:**
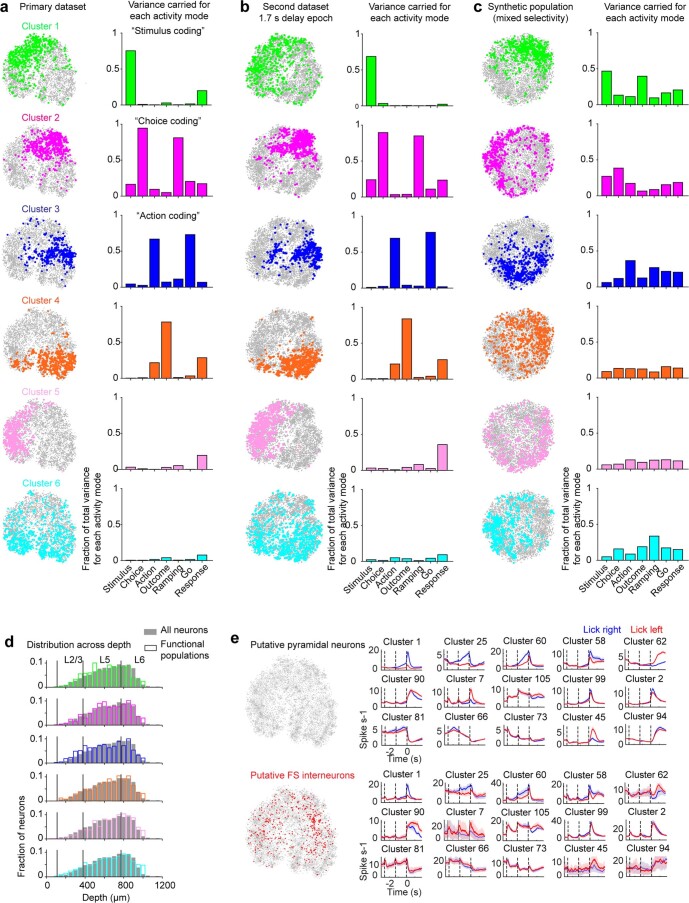
Functional populations. **a** Neurons in the primary dataset are divided into 6 functional populations using k-mean clustering on the weights of the 7 activity modes (Methods). *Left*, different functional populations in the t-SNE representation. *Right*, fraction of variance carried for each activity mode. For each activity mode, the fraction of variance across all 6 functional populations adds up to 1. Most functional populations carry the majority of variance for single activity modes. Functional populations that carry the most variance for the stimulus, choice, and action modes are termed stimulus, choice, and action coding. Choice coding population also carries most of the variance for the ramping mode. Action coding population also carries most of the variance for the go mode. The response mode is more evenly distributed across different functional populations. Only neurons with more than 5 error trials of each trial type are included (n = 3966). **b** Same as (**a**) but for the second dataset. n = 4010 neurons. **c** Same as (**a**) but for the synthetic population in which the coding of activity modes is randomly mixed. n = 3966 synthetic neurons. **d** Distribution of functional populations across depth. Open bars, distribution of specific functional populations; gray bars, distribution of all neurons in the dataset. Silicon probe recordings preferentially sample neurons from the deep layers. The distribution of each functional population does not differ from the distribution expected from sampling (gray). Neurons from the primary dataset. **e** *Left*, putative pyramidal neurons (gray dots) and fast-spiking interneurons (red dots) in the t-SNE representation. Interneuron responses are also diverse and span all prototypical response profiles observed in ALM. *Right*, average PSTHs of pyramidal neurons (top) and interneurons (bottom) in example clusters. Mean ± SEM across neurons. Interneurons exhibit similar PSTHs as the pyramidal neurons, but exhibit less trial-type selectivity.

These analyses show that distinct task selectivity was not randomly mixed across ALM populations. We used *k*-means clustering to divide neurons into functional populations based on their contribution to distinct activity modes (Fig. 3e, Extended Data Fig. 5a and Methods). Each functional population carried a majority of variance for specific activity modes while carrying little variance for other activity modes (Extended Data Fig. 5a). The choice coding population carried some variance for the stimulus and action modes, indicating partial overlaps with stimulus and action coding populations (Extended Data Fig. 5a,b). Nevertheless, the degree of un-mixing was substantial compared with the synthetic population coding random mixtures of selectivity (Extended Data Fig. 5c). The choice coding population also carried most of the variance for the ramping mode, further confirming their shared neural coding (Extended Data Fig. 5a,b). Stimulus, choice and action coding populations occupied distinct but partially overlapping locations in the *t*-SNE (Fig. 3e). A nearest-neighbor classifier could reliably classify stimulus, choice and action coding neurons based on their *t*-SNE locations (Fig. 3f). Thus, the functional populations can be delineated by their response profiles. The same pattern of clustering was also observed in the second dataset (Extended Data Fig. 5b). These data imply the progression from stimulus to choice and action activity unfolded across distinct but partially overlapping circuits instead of a single multiplexed circuit.

We did not find obvious anatomical separations between functional populations in their layer distributions (Fig. 3g and Extended Data Fig. 5d) or putative pyramidal neuron versus interneuron cell types (Extended Data Fig. 5e). We thus sought to relate different functional populations to specific long-range inputs to ALM.

### ALM receives long-range inputs from S1/S2, cALM and Thal_ALM_

**Fig. 4:**
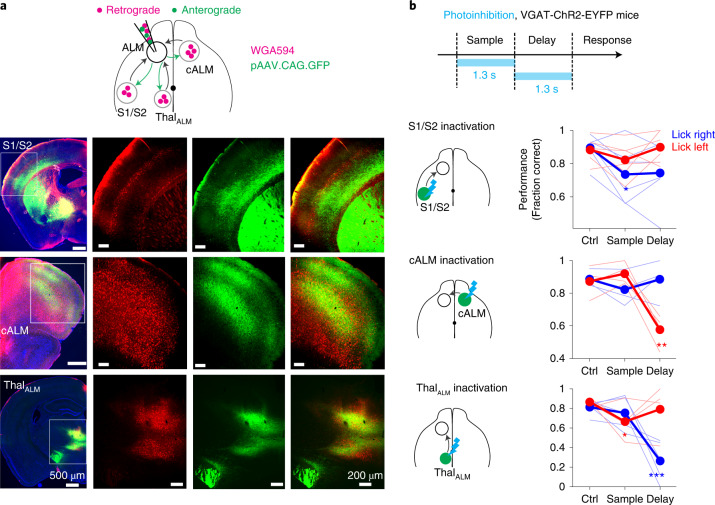
ALM receives long-range inputs from S1/S2, cALM and Thal_ALM_ which are required for behavior. **a**, Retrograde and anterograde tracing from ALM. Left: labeling in ipsilateral S1/S2, cALM and ipsilateral Thal_ALM_. Right: magnified images. Red, retrograde labeling (WGA-Alexa594); green, anterograde labeling (GFP); blue, Nissl stain. Retrograde and anterograde tracings were performed in the same brain. This experiment was repeated in five mice with similar results. **b**, Behavioral performance in the tactile decision task with photoinhibition of left S1/S2 (top), right ALM (middle) or left Thal_ALM_ (bottom) during different trial epochs. Thick lines, mean; thin lines, individual mice (S1/S2, *n* = 6; cALM *n* = 4; Thal_ALM_, *n* = 5). S1/S2, *P = 0.012; cALM, **P = 0.0016; Thal_ALM_, *P = 0.015, ***P = 0.0001; *P* values obtained by nested bootstrap across mice, sessions and trials, one-sided test (Methods). Ctrl, control.

Activity supporting the tactile decision is orchestrated by reciprocal interactions between ALM and connected brains regions^23,42^. Retrograde tracer injections (wheat germ agglutinin (WGA)) in ALM labeled ipsilateral somatosensory cortex, including parts of the primary and secondary somatosensory cortex (here collectively referred to as S1/S2); cALM; and ipsilateral thalamus, including parts of the ventral-medial nucleus, ventral-anterior-lateral nucleus (VAL), medial-dorsal nucleus and intralaminar nuclei (here collectively referred to as Thal_ALM_; Fig. 4a)^25^. Anterograde tracing shows that these regions were also targets of ALM projections (Fig. 4a). Thus, ALM formed reciprocal loops with ipsilateral S1/S2, cALM and ipsilateral Thal_ALM_.

We confirmed the involvement of S1/S2, cALM and Thal_ALM_ in the tactile decision behavior by optogenetically silencing these regions (Methods)^14,25,26^. Photoinhibition of the left S1/S2 (contralateral to the tactile stimulus) impaired task performance primarily during the sample epoch (Fig. 4b). Photoinhibition of cALM and Thal_ALM_ during the delay epoch biased upcoming lick direction to the ipsilateral direction (Fig. 4b). Photoinhibition of Thal_ALM_ during the sample epoch also impaired task performance (Fig. 4b). These results defined three input regions to ALM that causally contributed to the tactile decision behavior. We next examined the relative contributions of these inputs to task-related activity in ALM.

### Each long-range input connects to all response profiles

**Fig. 5:**
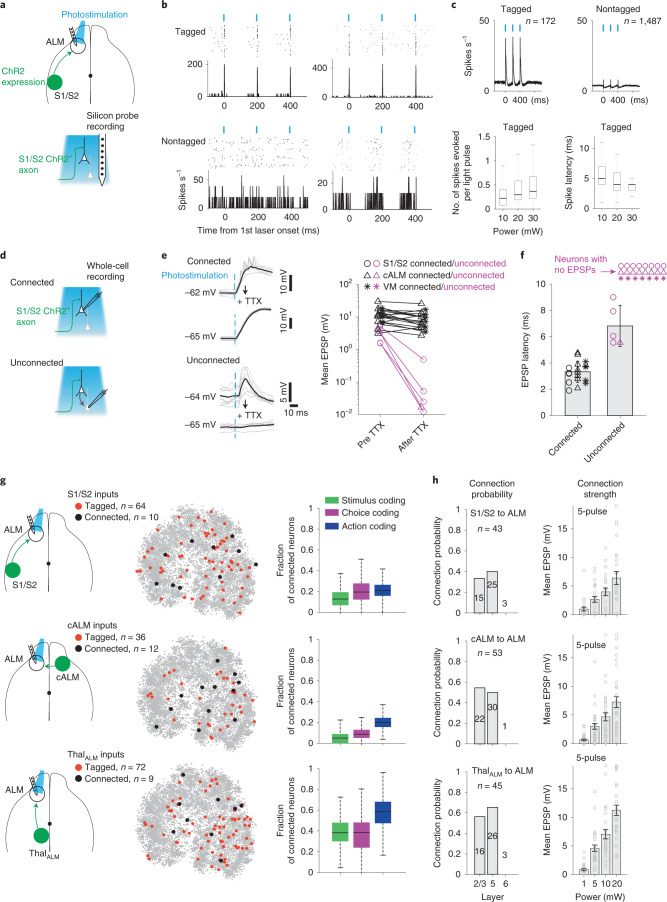
S1/S2, cALM and Thal_ALM_ inputs connect to all response profiles in ALM. **a**, Measuring long-range input connectivity using ChR2-tagging. **b**, Top: example neurons with short-latency responses to photostimulation of S1/S2 axons (tagged). Bottom: example ALM neurons unresponsive or suppressed by photostimulation (nontagged). Photostimulus, 1-ms pulses, 30 mW. **c**, Top: average response of tagged (*n* = 172) and nontagged neurons (*n* = 1,487). S1/S2, cALM and Thal_ALM_ axonal photostimulation data are combined. Bottom: response magnitude and latency of tagged neurons. Box and whisker plot shows median, 25/75th percentiles and most extreme data points not considered as outliers. **d**, ChR2-assisted circuit mapping. **e**, Calibration recordings from ALM. Left: example EPSPs. S1/S2 axonal photostimulation. Application of TTX left EPSP intact in a connected neuron (top). TTX abolished EPSP in an unconnected neuron (bottom). Right: mean EPSP before and after TTX for all tested neurons. Photostimulation power, 20 mW. **f**, EPSP latency of neurons verified to be connected or unconnected using TTX. Mean ± s.d. across neurons (S1/S2, *n* = 17; cALM, *n* = 17; Thal_ALM_, *n* = 13). A latency threshold (5 ms) could differentiate connected and unconnected neurons. Dots, individual neurons. Unconnected neurons with no EPSPs are shown on top. **g**, Left: ALM neurons connected to S1/S2 (top), cALM (middle) and Thal_ALM_ inputs (bottom) shown in the *t*-SNE. Colored dots, connected neurons measured from ChR2-tagging (red) and ChR2-assisted circuit mapping (black); gray dots, all neurons in the dataset. Only a subset of the neurons are tested for input connectivity. Right: fraction of connected neurons relative to all tested neurons within each functional population (Fig. 3e). Box and whisker plot shows median, 25/75th percentiles and most extreme data points not considered as outliers (bootstrap, Methods). **h**, S1/S2, cALM and Thal_ALM_ inputs differed in strength. Left: connection probability from ChR2-assisted circuit mapping. Numbers on each bar indicate the number of tested neurons. Right: light-induced EPSP in the connected neurons. Mean ± s.e.m. across neurons (dots). S1/S2, *n* = 43; cALM, *n* = 53; Thal_ALM_, *n* = 45. Only a subset of the neurons in panel **h** are tested in behavior, shown in panel **g**. See Extended Data Fig. 8. VM, ventral-medial nucleus.

We examined whether S1/S2, cALM and Thal_ALM_ preferentially innervated ALM neurons with certain response profiles by recording selectively from neurons postsynaptic to specific long-range inputs. We expressed ChR2 in ipsilateral S1/S2, cALM or ipsilateral Thal_ALM_ in separate groups of mice (Fig. 5a). During silicon probe recordings, we photoactivated ChR2-expressing axon terminals in ALM to activate postsynaptic neurons (‘ChR2-tagging’). Photostimulation (1-ms pulses) elicited time-locked responses in a small subset of neurons with short latency (Fig. 5b,c). The light-evoked response increased with photostimulus intensity while the response latency decreased (Fig. 5c). These neurons were deemed putative postsynaptic neurons to specific long-range inputs (Methods).

**Extended Data Fig. 6:**
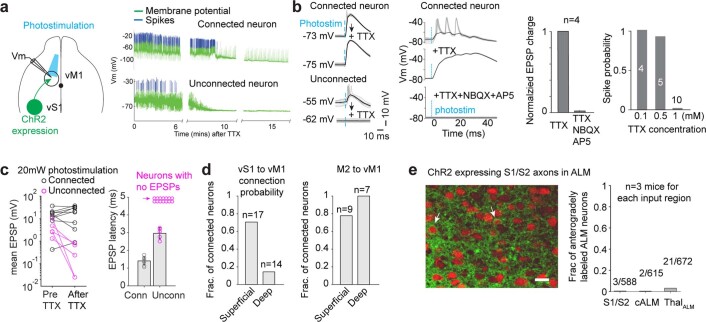
ChR2-assisted circuit mapping and validation. **a** *Left*, calibration whole-cell recordings measuring vS1→vM1 connectivity using ChR2-assisted circuit mapping. *Right*, recordings from two example vM1 neurons during photostimulation of vS1 axons. The traces show 15 mins of recordings after TTX application. Green, membrane potential; blue, action potentials. TTX left EPSPs intact in the connected neuron (*top*, see light-induced EPSPs in the green trace after action potentials were eliminated). TTX abolished EPSPs in the unconnected neuron (*bottom*). **b** Pharmacology to verify synaptic connections. *Left*, photostimulation of vS1 axons elicits short-latency EPSPs in vM1 neurons. Application of TTX left EPSPs intact in a connected neuron (*top*). TTX abolished EPSPs in an unconnected neuron (*bottom*). *Middle*, data from a connected neuron. Application of TTX left EPSPs intact. Application of AMPAR antagonist NBQX and NMDAR antagonist AP5 abolished the remaining EPSPs, confirming that the remaining EPSPs resulted from synaptic depolarization. Thin lines, individual photostimulation repetitions; thick lines, mean. *Right*, data from all recordings tested with TTX, NBQX and AP5. Neurons tested with TTX, NBQX and AP5, n = 4; neurons tested with various TTX concentrations, n = 19. **c** *Left*, mean EPSP before and after TTX for all tested vM1 neurons. *Right*, EPSP latency of all vM1 neurons verified to be connected or unconnected using TTX. Mean ± SD across neurons (n = 28 neurons). Dots, individual neurons. Photostimulation power, 20 mW. Unconnected neurons with no EPSPs are shown on top. The EPSP latencies in vM1 neurons are overall faster than ALM neurons (Fig. 5f). Nevertheless, connected and unconnected neurons could be differentiated based on latency. **d** *Left*, connection probability of vS1 inputs in vM1 superficial (<570 µm) and deep layers (>570 µm). vS1 inputs preferentially excite vM1 superficial layers, consistent with^24^. *Right*, connection probability of M2 inputs to vM1 inputs. M2 inputs preferentially excite vM1 deep layers, consistent with^24^. **e** Limited anterograde infection of ALM neurons from virus injections in S1/S2, cALM, and Thal_ALM_. *Left*, an example confocal image showing an ALM section 2 months after virus injection in S1/S2. Red, NeuN; green, ChR2 expression in S1/S2 input axons. Arrows indicate rare ALM neurons with ChR2 expression. *Right*, fraction of ALM neurons showing ChR2 or ReaChR expression for virus injections in S1/S2, cALM, and Thal_ALM_ (n = 3 mice each, 2–3 months after virus injection). The lack of ChR2 or ReaChR expression indicates that the light-induced EPSPs are due to ChR2 or ReaChR expressing long-range input axons. Scale bar, 10 µm.

Activation of ALM neurons could cause activity in locally connected neurons (Fig. 5d). We additionally applied ChR2-assisted circuit mapping^43^ in vivo to complement ChR2-tagging. During whole-cell recordings, we photoactivated ChR2-expressing axons from specific brain regions and we used short-latency excitatory postsynaptic potentials (EPSPs) to identify functional synapses. We performed calibration recordings in the vibrissa motor cortex (vM1) where the circuit connectivity has been well-mapped (Extended Data Fig. 6). Application of tetrodotoxin (TTX) abolished EPSPs in some neurons while leaving EPSPs intact in other neurons (Extended Data Fig. 6a,b). Blocking AMPA receptors and NMDA receptors with NBQX (6-nitro-2,3-dioxo-1,4-dihydrobenzo[f]quinoxaline-7-sulfonamide) and AP5 (2-amino-5-phosphonovalerate) abolished the remaining EPSPs, confirming that the response resulted from functional synapses (Extended Data Fig. 6b). At high photostimulation power (20 mW), EPSP latency could reliably distinguish connected neurons from unconnected neurons (Extended Data Fig. 6c). Using ChR2-assisted circuit mapping in vivo, we detected prevalent vibrissal somatosensory cortex (vS1) connections to the superficial layers of vM1 but not deep layers (Extended Data Fig. 6d), replicating the connectivity pattern measured in slices^20,21^. Calibration recordings and TTX pharmacology in ALM found that an EPSP latency threshold could also resolve S1/S2, cALM and Thal_ALM_ input connectivity (Fig. 5e,f). We thus used the latency threshold to distinguish postsynaptic neurons to specific long-range inputs.

**Extended Data Fig. 7:**
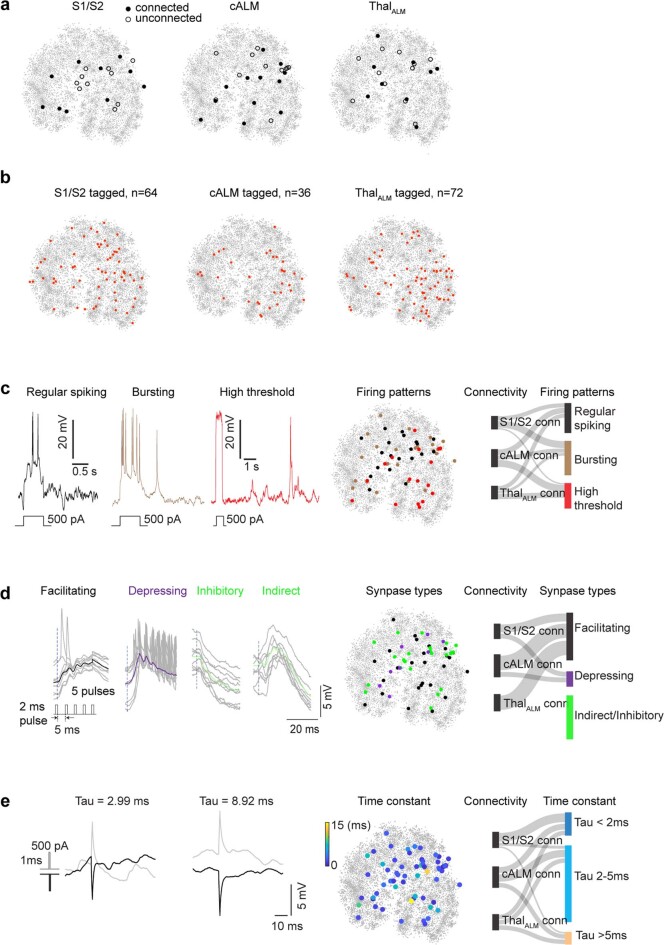
Connectivity and intrinsic properties of ALM neurons. **a** Connected (solid dots) and unconnected neurons (open dots) measured from ChR2-assist circuit mapping shown in the t-SNE representation. **b** Same as (**a**) but for connected neurons measured from ChR2-tagging. **c** Firing patterns of ALM neurons do not predict long-range input connectivity. *Left*, example firing patterns in response to current injections during whole-cell recording. Neurons are classified into regular spiking, bursting, and high threshold types (Methods). *Middle*, regular spiking (black), bursting (brown), and high threshold neurons (red) shown in the t-SNE representation. *Right*, classification flow chart of connected neurons to regular spiking, bursting, and high threshold types. **d** EPSP dynamics of ALM neurons do not predict long-range input connectivity. *Left*, example light-induced EPSPs. Neurons are classified into facilitating, depressing, and indirect/inhibitory types based on EPSP dynamics. *Middle*, neurons with facilitating (black), depressing (purple), and indirect/inhibitory responses (green) in the t-SNE representation. *Right*, classification flow chart of connected neurons to facilitating, depressing, and indirect/inhibitory responses. **e** Membrane time constant of ALM neurons does not predict long-range input connectivity. *Left*, example neurons with short and long time constant. Tau is measured as the time when EPSP decays to 67% of its peak amplitude after a 1-ms depolarizing or hyperpolarizing current injection. *Middle*, neurons with different time constant in the t-SNE representation. *Right*, classification flow chart of connected neurons to different time constant.

**Extended Data Fig. 8:**
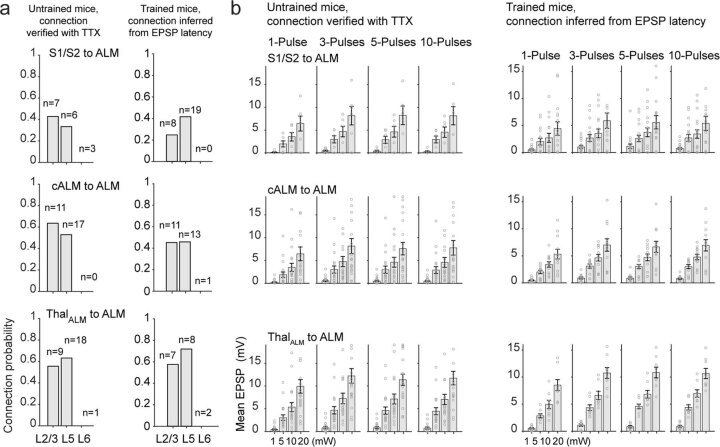
Connectivity patterns of S1/S2, cALM, and Thal_ALM_ inputs. **a** Connection probability across depth measured from whole-cell recording and ChR2-assisted circuit mapping. *Left*, data from untrained mice, in which connections are verified with TTX pharmacology. *Right*, data from mice trained in the tactile decision task, in which connections are inferred by EPSP latency. Only a subset of the neurons was held long enough to be tested further in the tactile decision task (Fig. 5g and S7). Numbers on each bar indicate the number of tested neurons. **b** Connection strength measured from whole-cell recordings. Mean ± SEM across neurons (each dot represents one neuron). Untrained mice, S1/S2, n = 16 neurons; cALM, n = 28 neurons; VM, n = 28 neurons. Trained mice, S1/S2, n = 27 neurons; cALM, n = 25 neurons; VM, n = 17 neurons. A range of photostimulation power (1, 5, 10, 20 mW) and pulse conditions (1, 3, 5, 10 pulses; 2-ms pulses at 5-ms interval) were tested. Photostimulation of Thal_ALM_ axons elicits the strongest EPSP (between S1/S2 or cALM and VM, *P* = 1.86 × 10^−8^, untrained group; *P* = 8.82 × 10^−14^, trained group, two-way ANOVA, powers and inputs).

S1/S2, cALM and Thal_ALM_ inputs indiscriminately targeted ALM neurons regardless of their response profiles (Fig. 5g and Extended Data Fig. 7a,b). Functional populations coding stimulus, choice and action were coupled to all three inputs (Fig. 5g; *P* > 0.05, chi-squared test, pair-wise comparisons across all functional populations). Connected neurons also exhibited similar intrinsic and synaptic properties (Extended Data Fig. 7c–e). Inputs from S1/S2, cALM and Thal_ALM_ differed in connection strength. Connection probability was higher for Thal_ALM_ inputs compared with S1/S2 inputs (Fig. 5h and Extended Data Fig. 8a; *P* < 0.05, chi-squared test, connection probability across all layers). Photostimulation of Thal_ALM_ axons also elicited stronger EPSPs than S1/S2 and cALM (Fig. 5h and Extended Data Fig. 8b; *P* < 0.001 for inputs, two-way analysis of variance (ANOVA) across inputs and photostimulation power).

These results show that S1/S2, cALM and Thal_ALM_ inputs targeted all response profiles in ALM. Long-range inputs mainly differed in strength: thalamic inputs provided the strongest excitatory drive to ALM compared with cortico-cortical inputs.

### Thalamus strongly influences all functional populations in ALM

To assess the contributions of S1/S2, cALM and Thal_ALM_ inputs to ALM activity, we silenced each brain region while recording from ALM. In VGAT-ChR2-EYFP mice, we placed an optical fiber above S1/S2, cALM or Thal_ALM_ (Methods). In S1/S2 and cALM, photostimulation excited ChR2 in local interneurons and inhibited nearby pyramidal neurons^44^, including neurons projecting to ALM. In Thal_ALM_, photostimulation excited the reticular nucleus axon terminals in Thal_ALM_ and silenced thalamic output^25,45^.

**Fig. 6:**
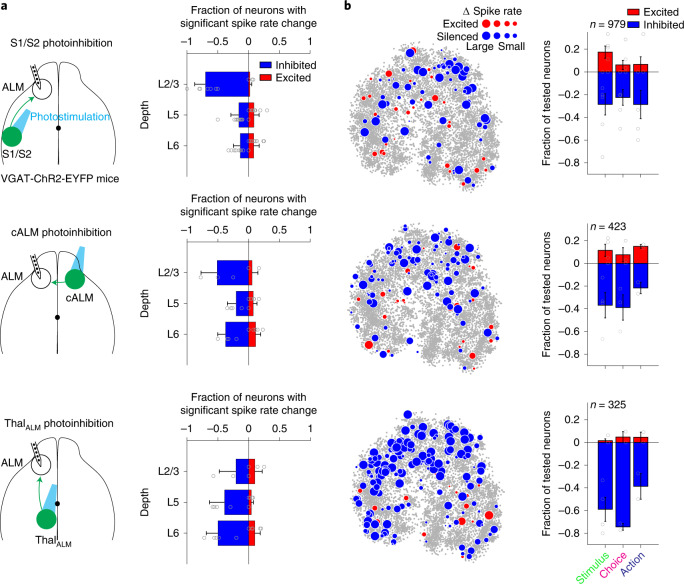
Thalamic inputs drive all response profiles in ALM. **a**, Effects of photoinhibiting S1/S2 (top), cALM (middle) and Thal_ALM_ (bottom) on ALM spike rates. Neurons are tested for significant spike rate change (Methods). Data from sample and delay epoch photoinhibition are combined. ‘Lick left’ and ‘lick right’ trials are pooled. Mean ± s.e.m. across mice (dots). S1/S2 photoinhibition, layer 2/3, *n* = 55 neurons; layer 5, *n* = 543; layer 6, *n* = 383, 12 mice. cALM photoinhibition, layer 2/3, *n* = 27; layer 5, *n* = 243; layer 6, *n* = 174, 10 mice. Thal_ALM_ photoinhibition, layer 2/3, *n* = 15; layer 5, *n* = 193; layer 6, *n* = 169, 9 mice. **b**, Effects of photoinhibiting S1/S2 (top), cALM (middle) or Thal_ALM_ (bottom) on ALM functional populations. Left: excited (red dots) or silenced (blue dots) neurons shown in the *t*-SNE. Dot size represents the magnitude of spike rate change during photoinhibition relative to control. Gray dots, all neurons in the dataset. Only a subset of the neurons are tested for photoinhibition. Right: fraction of excited and inhibited neurons relative to all tested neurons within each functional population. Only neurons with spike rates greater than 0.5 and tested for more than five error trials of each trial type are included. S1/S2 photoinhibition: *n* = 59, 63, 32 neurons, 12 mice, for stimulus, choice and action coding populations; cALM photoinhibition, *n* = 51, 56, 23 neurons, 10 mice; Thal_ALM_ photoinhibition, *n* = 44, 47, 18 neurons, 9 mice. Mean ± s.e.m. across mice (dots). Also see Extended Data Fig. 9. L, layer.

Photoinhibition of S1/S2 and cALM produced varied effects across cortical layers: most neurons in the superficial layers were silenced by S1/S2 and cALM photoinhibition, but the activity in the deep layers was less affected (Fig. 6a; *P* < 0.001 for both S1/S2 and cALM photoinhibition, one-way ANOVA). In contrast, Thal_ALM_ photoinhibition reduced ALM activity across all layers, with the strongest effect in the deep layers (Fig. 6a; *P* < 0.001, one-way ANOVA).

Silencing each input region impacted all response profiles. Photoinhibiting S1/S2, cALM or Thal_ALM_ depressed ALM neurons coding stimulus and choice (Fig. 6b). Neurons coding the action mode have low spike rates during the sample and delay epochs. In these experiments, photoinhibition was during the sample or delay epoch, and thus produced limited spike rate decrease in this population. Nevertheless, silencing each input region also reduced spike rate in action coding neurons (Fig. 6b). The broad effect of photoinhibition mirrored the connectivity of the long-range inputs, which contacted all functional populations (Fig. 5g). The effect of photoinhibition only differed in strength: silencing Thal_ALM_ inhibited a larger fraction of ALM neurons than silencing S1/S2 or cALM (Fig. 6b; Thal_ALM_ versus S1/S2 photoinhibition, *P* < 0.01 for stimulus and choice coding neurons, *P* = 0.88 for action coding neurons; Thal_ALM_ versus cALM photoinhibition, *P* < 0.01 for stimulus and choice coding neurons, *P* = 0.61 for action coding neurons, chi-squared test).

**Extended Data Fig. 9:**
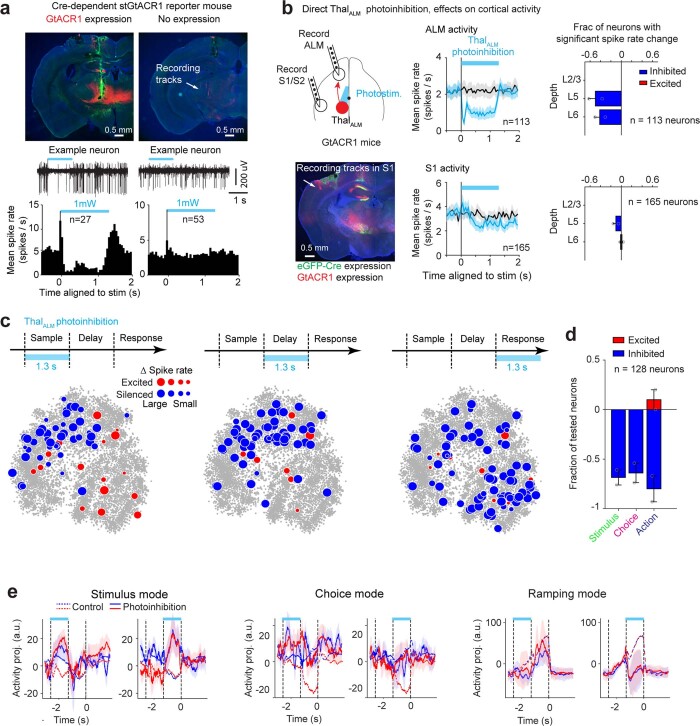
Direct Thal_ALM_ photoinhibition using GtACR1. **a** Direct photoinhibition of Thal_ALM_ using GtACR1. *Top*, injection of AAV Cre virus in Thal_ALM_ of a Cre-dependent GtACR1 reporter mouse. Green, expression of eGFP-Cre. Red, GtACR1 expression. Autofluorescence around optical fiber track is visible in green. After mice were tested in Thal_ALM_ photoinhibition, optical fiber implants were removed. Optrode recordings were made in Thal_ALM_ (left) and posterior thalamus (right) to characterize the spatial specificity of Thal_ALM_ photoinhibition. For posterior thalamus, recording tracks were labeled with DiI (arrow in the right image). Recordings were performed in awake non-behaving mice (n = 2). *Bottom*, example voltage traces and average activity of neurons in Thal_ALM_ and posterior thalamus. Photostimulation power, 1 mW. **b** Effect of direct Thal_ALM_ photoinhibition on cortical activity. *Left column*, silicon probe recordings in ALM (coordinates from bregma: anterior 2.5 mm, lateral 1.5 mm; n = 113, 2 mice) and S1 (posterior 1.0 mm, lateral 3.1 mm; n = 165, 2 mice). The image shows expression of eGFP-Cre (green) and GtACR1 (red) in Thal_ALM_. Recording tracks were labeled with DiI (arrow). *Middle column*, Thal_ALM_ photoinhibition reduced the spike rate in ALM, but not in S1. Average spike rates aligned to photostimulation. Black, control; cyan, photostimulation. Mean ± SEM across neurons. *Right column*: neurons with significant spike rate change, defined as *P* < 0.01 using two-tailed t-test between photoinhibition and control trials. Layer 2/3, 110–378 µm (ALM, n = 5 neurons; S1, n = 8); layer 5, 378–772 µm (ALM, n = 45; S1, n = 57); layer 6, below 772 µm (ALM, n = 63; S1, n = 100; Methods). Recordings were performed in awake non-behaving mice. Only neurons with spike rate above 1 Hz are included in the analysis. Mean ± SEM (bootstrap across neurons). Data from 2 mice, the fractions for individual mice are shown as dots. **c** Effect of direct Thal_ALM_ photoinhibition on ALM functional populations. Excited (red dots) or silenced (blue dots) neurons shown in the t-SNE representation. Gray dots, all neurons in the dataset (Fig. 1a). Only a subset of the neurons in the dataset are tested for photoinhibition. Photoinhibition is during the sample, delay, or response epoch. Action coding neurons have low spike rates during the sample and delay epoch. Thus photoinhibition during the sample and delay epoch induced limited silencing in this population (Fig. 6b). Here, response epoch photoinhibition strongly silenced action coding neurons. **d** Distribution of excited and inhibited neurons across functional populations. Fraction is relative to the total number of tested neurons within each functional population. Fraction for stimulus and choice coding populations are from sample and delay epoch photoinhibition. Fraction for action coding population is from response epoch photoinhibition. Mean ± SEM (bootstrap across neurons). Data from 2 mice, the fractions for individual mice are shown as dots. When functional populations are tested during the epoch in which they are active, the fraction of silenced neurons is similar. Stimulus vs. choice coding, *P* = 0.71; choice vs. action coding, *P* = 0.36; stimulus vs. action coding, *P* = 0.48, two-sided chi-square test. **e** Effect of direct Thal_ALM_ photoinhibition on ALM activity modes. Mean ± SEM (bootstrap, Methods). Dotted lines show activity projections of control trials. The effects are similar to Fig. 7.

Thal_ALM_ photostimulation in VGAT-ChR2-EYFP mice may activate GABAergic axons of the thalamic reticular nucleus or substantia nigra pars reticulata, which may inhibit other thalamic nuclei. We directly inhibited Thal_ALM_ using a light-dependent chloride channel (GtACR1, Methods)^46^. GtACR1 was expressed around thalamic ventral-medial nucleus by injecting AAV Cre in a Cre-dependent GtACR1 reporter mouse^44^ (Extended Data Fig. 9a and Methods). Silicon probe recordings in thalamus and cortex confirmed that photoinhibition was limited to Thal_ALM_ (Extended Data Fig. 9a,b). Direct Thal_ALM_ photoinhibition strongly inhibited ALM activity, primarily in the deep layers (Extended Data Fig. 9b). We inhibited Thal_ALM_ during all task epochs, including the response epoch when action coding neurons were active (Extended Data Fig. 9c). When activity level was accounted for, Thal_ALM_ photoinhibition equally suppressed stimulus, choice and action coding neurons (Extended Data Fig. 9d; *P* > 0.05, chi-squared test, pair-wise test across all populations).

These results show that S1/S2, cALM and Thal_ALM_ inputs each influenced all functional populations in ALM. Cortico-cortical projections affected superficial layers more than deep layers. Thalamic inputs affected deep layers more than superficial layers, and thalamic inputs affected more total neurons in ALM than cortico-cortical inputs.

### Activity coding stimulus, choice and action requires thalamic inputs

Finally, we examined the contributions of S1/S2, cALM and Thal_ALM_ inputs to distinct activity modes coding behavior.

**Fig. 7:**
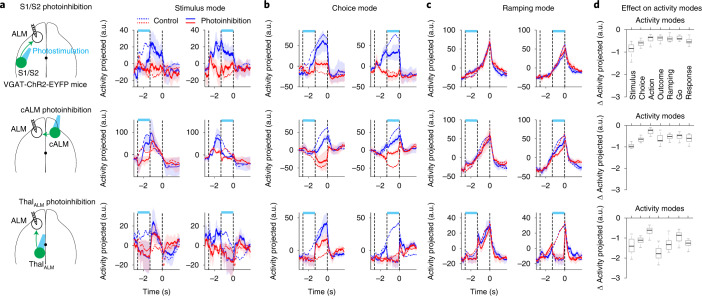
Activity modes coding stimulus and choice require thalamic inputs. **a**, Effects of photoinhibiting S1/S2 (top), cALM (middle) and Thal_ALM_ (bottom) on ALM stimulus mode. Mean ± s.e.m. (bootstrap, Methods). Dotted lines, activity in control trials. Dashed lines delineate behavioral epochs. Both correct and error trials are included in activity projections, grouped by instructed trial type. Blue, ‘lick right’ trials; red, ‘lick left’ trials. **b**, Same as **a** but for ALM choice mode. Mean ± s.e.m. (bootstrap, Methods). **c**, Same as **a** but for ALM ramping mode. Mean ± s.e.m. (bootstrap, Methods). **d**, Changes in ALM activity modes during photoinhibition relative to control trials. Activity changes in ‘lick left’ and ‘lick right’ trials are averaged. Data from sample and delay epoch photoinhibition are combined. Only neurons with more than five error trials and at least two photoinhibition trials for each trial type are included (S1/S2, *n* = 326; cALM, *n* = 310; Thal_ALM_, *n* = 208). Box and whisker plot shows median, 25/75th percentiles and most extreme data points not considered as outliers. Thal_ALM_ versus S1/S2, *P* = 0.039, 0.001, 0.11, <1 × 10^−4^, 0.003, 0.025, 0.011 for stimulus, choice, action, outcome, ramping, go, response modes, respectively; Thal_ALM_ versus cALM, *P* = 0.065, 0.016, 0.059, 0.003, 0.005, 0.098, 0.019. *P* values obtained by bootstrap, one-sided test (Methods).

Photoinhibiting S1/S2 during the sample epoch depressed ALM stimulus mode, consistent with S1/S2 providing stimulus information to ALM. However, trial-type selectivity recovered after S1/S2 photoinhibition (Fig. 7a), and thus the stimulus information was not completely lost despite blocking S1/S2 transmission. These observations are in line with the relatively mild effect of S1/S2 photoinhibition on behavioral performance (Fig. 4b). This suggests that stimulus information could reach ALM through other pathways that were not blocked by the photoinhibition. Photoinhibiting S1/S2 during the delay epoch minimally affected ALM stimulus mode (Fig. 7a), further indicating that ALM could maintain stimulus information in absence of S1/S2 inputs.

Photoinhibiting Thal_ALM_ during the sample epoch also collapsed ALM stimulus mode. In contrast to S1/S2 photoinhibition, trial-type selectivity did not recover after Thal_ALM_ photoinhibition (Fig. 7a). Thus, blocking thalamic transmission permanently abolished stimulus information in ALM. Photoinhibiting Thal_ALM_ during the delay epoch also collapsed ALM stimulus mode. These data show that ALM stimulus selectivity required thalamic inputs.

ALM choice mode was also affected by S1/S2 and cALM photoinhibition (Fig. 7b). However, Thal_ALM_ photoinhibition totally collapsed ALM choice selectivity. Thal_ALM_ photoinhibition during the sample epoch persistently depressed choice selectivity during the delay epoch (Fig. 7b). This is consistent with the reduction in task performance induced by sample epoch Thal_ALM_ photoinhibition (Fig. 4b), which suggests that thalamic inputs during the sample epoch were required for generating correct choice. Thal_ALM_ photoinhibition during the delay epoch collapsed choice selectivity (Fig. 7b), confirming previous reports that thalamic inputs are required to maintain choice selective delay activity^25^.

The nonselective ramping activity was resistant to S1/S2 and cALM photoinhibition (Fig. 7c). Thus, ALM ramping activity could reflect signals generated outside of the neocortex. Thal_ALM_ photoinhibition during either the sample or delay epoch suppressed ALM ramping activity (Fig. 7c). Interestingly, after Thal_ALM_ photoinhibition during the sample epoch, ramping activity recovered to the trajectory of unperturbed trials and continued uninterrupted (Fig. 7c). This further indicates that the ramping signal was generated outside of the frontal thalamocortical loop and was transmitted through the thalamus to ALM.

**Extended Data Fig. 10:**
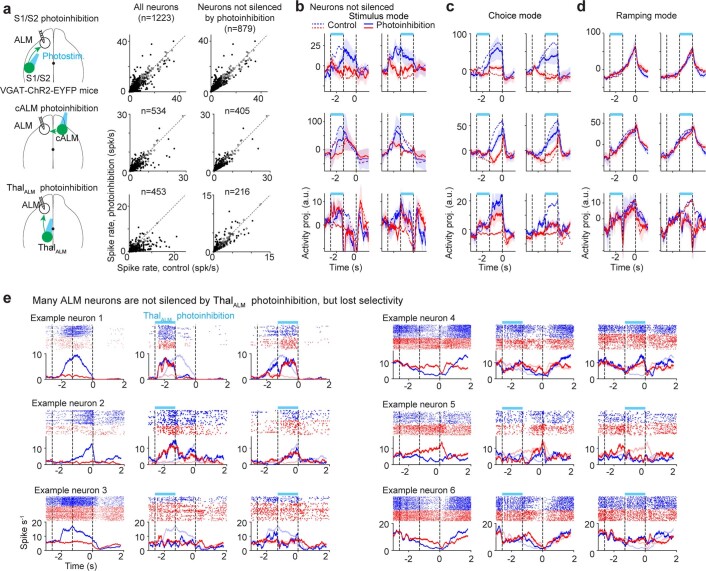
Effects of S1/S2, cALM, and Thal_ALM_ photoinhibition on ALM activity. **a** Spike rates during photoinhibition (1.3 s) versus control conditions. Filled circles, ALM neurons that are significantly modulated by photoinhibition (defined as *P* < 0.01 using a two-tailed t-test between photoinhibition and control trials). *Left column*, all neurons. *Right column*, neurons that retained >50% of the control condition spike rate during photoinhibition. **b** Effects of photoinhibition on ALM stimulus mode. Activity modes are computed using only neurons that retained >50% of control condition spike rate during photoinhibition. Mean ± SEM (bootstrap, Methods). Dotted lines show activity projections of control trials. Dashed lines delineate behavioral epochs. The effects are similar to Fig. 7. **c** Same as (**b**) but for ALM choice mode. Mean ± SEM (bootstrap, Methods). **d** Same as (**b**) but for ALM ramping mode. Mean ± SEM (bootstrap, Methods). **e** Example ALM neurons during Thal_ALM_ photoinhibition. Spike raster and PSTH. *Left*, control trials; *middle*, sample epoch photoinhibition; *right*, delay epoch photoinhibition. Cyan bars, photostimulation period. Neurons are not silenced by Thal_ALM_ photoinhibition, but lost selectivity.

Across all activity modes, silencing Thal_ALM_ produced a greater effect than silencing S1/S2 and cALM (Fig. 7d). We examined whether thalamic inputs drove stimulus and choice selectivity or simply provided excitatory drive to maintain spike rates in ALM without conveying task-related information. First, we noted that Thal_ALM_ photoinhibition in our experiments induced only moderate reduction in ALM spike rate: 37.8% of ALM neurons showed a spike rate reduction of 50% or more while 9% of ALM neurons showed a significant increase in spike rate (Extended Data Fig. 10a; *P* < 0.01, two-tailed *t*-test, photoinhibition versus control). Even among the neurons that did not show a decrease in spike rate, Thal_ALM_ photoinhibition still resulted in collapsed activity modes (Extended Data Fig. 10b–d). Finally, selectivity was abolished in individual neurons even when spike rates were little affected (Extended Data Fig. 10e). These data suggest that thalamic inputs drive ALM task selectivity.

**Fig. 8:**
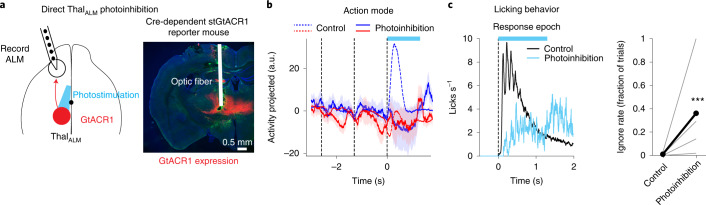
Action mode and movement initiation require thalamic inputs. **a**, Direct photoinhibition of Thal_ALM_ using GtACR1. Injection of AAV Cre virus in a Cre-dependent GtACR1 reporter mouse. Red, expression of GtACR1 in Thal_ALM_. This experiment was repeated in seven mice with similar results. **b**, Effect of photoinhibiting Thal_ALM_ on ALM action mode. Dotted lines, activity in control trials. Dashed lines delineate behavioral epochs. Correct, error and ignore trials are combined, grouped by instructed trial type. Blue, ‘lick right’ trials; red, ‘lick left’ trials. Photostimulation power, 1–3 mW. **c**, Left: lick rate in control and Thal_ALM_ photoinhibition trials (*n* = 5 mice). Right: fraction of ignore trials in control and Thal_ALM_ photoinhibition trials. Thick lines, mean; thin lines, individual mice. ***P < 1 × 10^−4^; *P* value obtained by nested bootstrap across mice, sessions and trials, one-sided test (Methods).

Silencing Thal_ALM_ also affected the action mode (Fig. 7d). However, the early experiment only tested photoinhibition during the sample and delay epochs. In a separate experiment, we directly silenced Thal_ALM_ during the response epoch using GtACR1 (Fig. 8a and Extended Data Fig. 9). ALM action mode was abolished by Thal_ALM_ photoinhibition (Fig. 8b). With Thal_ALM_ photoinhibition, mice frequently did not initiate licking after the ‘go’ cue (Fig. 8c). These findings are consistent with previous reports that ALM activity is necessary for initiating licking^14,35,37^, and they further reveal an indispensable role of thalamic inputs.

In sum, these data show that both cortico-cortical and thalamic inputs contributed to ALM task selectivity during decision-making, but thalamic inputs had the strongest impact. Thus, ALM task selectivity required thalamic inputs.

## Discussion

Our analysis in mouse ALM uncovers highly organized activity during tactile decision-making. Individual neurons exhibit a collection of prototypical response profiles that are repeatable across mice (Fig. 1), which implies a structured underlying circuit. Contrary to a shared circuit that multiplexes different task selectivity, activity coding stimulus, choice and action unfolds across distinct but partially overlapping functional populations that can be delineated by their response profiles (Fig. 3). Each functional population receives inputs from somatosensory cortex (S1/S2), cALM and thalamus (Fig. 5). Both cortico-cortical and thalamic inputs contribute to task selectivity, but thalamic inputs have the strongest impact (Figs. 6 and 7). Our results suggest a model in which thalamic inputs drive distinct subnetworks within frontal cortex coding different features of behavior.

Despite direct cortico-cortical connections between S1/S2 and ALM, stimulus selectivity in ALM is dependent on thalamic inputs. S1/S2 inputs provide only weak excitation to ALM and do not preferentially target stimulus coding neurons (Figs. 5g and 6). Blocking S1/S2 transmission only transiently reduced stimulus information in ALM, while leaving stimulus information intact during the delay epoch (Fig. 7a). In contrast, blocking thalamic transmission completely and persistently abolished stimulus information in ALM (Fig. 7a). Together, these data suggest redundant subcortical pathways signal stimulus information to ALM via the thalamus.

Previous studies found that thalamic inputs are required to maintain choice selective persistent activity in frontal cortex during the delay epoch^25,47,48^. Thalamus relays information from subcortical loops to frontal cortex^49–51^. Our study extends these findings by revealing distinct sources of selectivity supporting different aspects of decision-making. Transiently blocking thalamic transmission during the sample epoch persistently disrupted choice selectivity (Fig. 7) and impaired task performance (Fig. 4b), suggesting that choice formation depends on thalamocortical transmission. Our side-by-side comparisons with cortico-cortical projections further uncover that thalamus drives ramping activity coding an urgency signal (Fig. 7). Ramping remained intact after transiently blocking thalamic transmission, and thus ramping activity appears to originate outside of the thalamocortical loop^49,52,53^. Finally, activity related to licking action requires thalamic inputs (Fig. 8), consistent with recent reports that thalamic inputs to frontal cortex are required for movement initiation^35,54,55^.

Our thalamus photoinhibition only partially reduced ALM activity. Yet selectivity was abolished, even in neurons showing no change or increased spike rates (Extended Data Fig. 10). These data suggest the thalamus directly drives selectivity in ALM. Silencing S1/S2 and cALM induced non-negligible effects on the stimulus and choice modes. More work is needed to resolve the interaction of thalamic inputs with cortical inputs^48^.

Previous recordings in rodent frontal and parietal cortex found randomly mixed selectivity for stimulus, choice and action within shared neuronal populations^1,7,9^. Our analyses show that segregations exist: stimulus coding neurons are less likely to encode choice or action and vice versa, with some overlap (Fig. 3d). These results are potentially consistent with findings in learned recurrent neural networks showing neurons with similar selectivity tend to form functional subnetworks^56^. In the tactile decision task, the addition of a delay epoch may have facilitated the separation of different neural coding. Differences in brain areas may also explain some differences. Finally, it is possible that structures in population activity only become apparent with sufficiently large neuronal samples. Our power analysis shows that at least 400 neurons are needed to detect structures in neural coding (Extended Data Fig. 4c,d), and some ALM neurons did exhibit mixtures of selectivity (Fig. 3d).

We find that choice and ramping signals are coded by a shared neuronal population (Fig. 3d). Previous modeling suggests that ramping signal plays a permissive role for choice selectivity to develop^27,30^. Multiplexed choice and ramping signals in a shared population may enable such interactions to occur. Our results do not rule out mixed selectivity across modalities within the stimulus and choice coding populations. For example, during multi-sensory decision-making, the same population could multiplex information from multiple sensory modalities^9^. It will be of interest to determine if the same stimulus and choice coding populations encode stimulus and choice across tasks and modalities.

We find that the majority of ALM neurons conform to a fixed repertoire of prototypical response profiles under similar task conditions, and defined neuronal populations contribute to specific neural coding. A small proportion of ALM neurons could not be reliably assigned to a response profile cluster (Extended Data Fig. 1d,e). This could be due to insufficient number of neurons to define small clusters or poor estimates of response profiles from limited number of spikes. Idiosyncratic differences between mouse behaviors may also contribute to irregular response profiles. It remains to be determined if ALM responses fully conform to a finite set of discrete clusters. A related question is whether ALM functional populations receive like-to-like versus nonspecific thalamic connections^57,58^. Finding organized activity in frontal cortex paves the way for linking behavior-related signals to detailed thalamocortical connectivity^22^.

## Methods

### Mice

This study was based on data from 186 mice (age > postnatal day 60, both male and female mice). Sixteen VGAT-ChR2-EYFP mice (JAX 014548), four PV-ires-cre (JAX 008069)^59^ crossed with a red-shifted channelrhodopsin (ReaChR) reporter mice (Rosa26-LSL-ReaChR-mCitrine, JAX 026294)^60^ and one PV-ires-cre mouse crossed with Ai32 (Rosa26-LSL-ChR2-EYFP, JAX 012569) were used for electrophysiology and photoinhibition during behavior experiments. Seven GtACR1 reporter mice (R26-LNL-GtACR1-Fred-Kv2.1, JAX 033089) were used for Thal_ALM_ photoinhibition^44^. Seventeen Ai32 mice were used for in vivo whole-cell recording to characterize connectivity from vS1 and secondary motor cortex (M2) to vM1. Twenty-eight ReaChR mice were used for in vivo whole-cell recording to characterize S1/S2, cALM and Thal_ALM_ connectivity to ALM in untrained passive mice. Sixteen ReaChR and 11 Ai32 mice were used for in vivo whole-cell recording of S1/S2, cALM and Thal_ALM_ connectivity to ALM during tactile decision-making behavior. Ten ReaChR mice were used for silicon probe recording and ChR2-tagging to characterize S1/S2, cALM and Thal_ALM_ connectivity to ALM during behavior experiments. Finally, five wildtype mice were used for anatomical tracing.

We analyzed three silicon probe recording datasets previously collected in the same task conditions (from refs. ^14,24,31^). Combined, the primary extracellular recording dataset (1.3-s delay epoch) included new data from 31 mice (described above, 4,967 units) and reused data from 42 mice (4,659 units)^14,24^. The second extracellular recording dataset (1.7-s delay epoch) included reused data from refs. ^24,31^ (29 mice, 10,420 neurons).

All procedures were in accordance with protocols approved by the Institutional Animal Care and Use Committees at Baylor College of Medicine. Mice were housed at a constant temperature (22 ± 1 °C) and humidity (30–55%) under a 12:12 reversed light/dark cycle and tested during the dark phase. On days not tested, mice received 0.5–1 ml of water. On other days, mice were tested in experimental sessions lasting 1–2 h where they received all their water (0.3–1 ml). If mice did not maintain a stable body weight, they received supplementary water^61^. All surgical procedures were carried out aseptically under 1–2% isoflurane anesthesia. Buprenorphine Sustained Release (1 mg kg^−1^) and Meloxicam Sustained Release (4 mg kg^−1^) were used for preoperative and postoperative analgesia. A mixture of bupivacaine and lidocaine was administered topically before scalp removal. After surgery, mice were allowed to recover for at least 3 d with free access to water before water restriction.

### Surgery

Mice were prepared with a clear skull-cap and a headpost^26,61^. The scalp and periosteum over the dorsal skull were removed. A layer of cyanoacrylate adhesive (Krazy glue, Elmer) was directly applied to the intact skull. A custom headpost was placed on the skull over the visual cortex and cemented in place with clear dental acrylic (Lang Dental Jet Repair Acrylic; Part no. 1223-clear). A thin layer of clear dental acrylic was applied over the cyanoacrylate adhesive covering the entire exposed skull, followed by a thin layer of clear nail polish (Electron Microscopy Sciences, Part no. 72180). For Thal_ALM_ photoinhibition, a 5-mm optic fiber (Thorlabs, Part no. CFMLC12L05) was implanted above the left Thal_ALM_^25^. For cALM or S1/S2 photoinhibition, a plastic fitting was glued onto the clear skull implant above the right ALM or left S1/S2 for attachment of the optic fiber.

### Behavior

The behavioral task and training have been described^61,62^. The stimulus was a metal pin (0.9 mm in diameter), presented at one of two possible positions (Fig. 1a). The two pole positions were 5 mm apart along the anterior–posterior axis. The posterior pole position was 5 mm from the whisker pad. A two-spout lickport (4.5 mm between spouts) was used to deliver water rewards and record licks.

At the beginning of each trial, the vertical pole moved into reach of the whiskers (0.2-s travel time), where it remained for 1 s, after which it was retracted (retraction time 0.2 s). The sample epoch was defined as the time between the pole movement onset to 0.1 s after the pole retraction onset (sample epoch, 1.3 s; Fig. 1a). Mice touched the object at both pole positions, typically with a different set of whiskers. The delay epoch (1.3 s for primary dataset, 1.7 s for second datasets) followed the sample epoch. An auditory ‘go’ cue indicated the end of the delay epoch (pure tone, 3.4 kHz, 0.1 s). Licking early during the trial was punished by a loud alarm sound (0.05 s) and a brief timeout (1–1.2 s). Licking the correct lickport after the ‘go’ cue led to a water reward (2–3 µl). Licking the incorrect lickport triggered a timeout (2–6 s). Trials in which mice did not lick within a 1.5-s window after the ‘go’ cue (‘ignore’) were rare and typically occurred at the end of a session. Reaction time was from the ‘go’ cue onset to the first lickport contact.

### Viral injection and histology

Glass pipettes (20–30-µm-diameter tip and beveled) were back-filled with mineral oil and front-loaded with viral suspension immediately before injection.

For anatomical tracing, AAV2.CAG.GFP (Addgene, 37825) was injected in the left ALM (anterior 2.5 mm from lambda, lateral 1.5 mm, depth 0.5 and 0.8 mm, 100 nl at each depth). At 14 d post injection, WGA (Thermo Fisher Scientific, WGA-Alexa594, 2% in PBS, 200 nl) was injected in the same location and incubated for 24 h. Mice were perfused transcardially with PBS followed by 4% paraformaldehyde (PFA)/0.1 M PBS. The brains were fixed overnight in 4% PFA and transferred to 30% sucrose before sectioning on a cryostat (Thermo Scientific, Cryostar NX70). Coronal 30-µm free-floating sections were mounted with mounting medium with DAPI (Vector Laboratories, H-1500-10), imaged on a fluorescence macroscope (Olympus MVX10) and processed in ImageJ.

To characterize long-range input connectivity in ALM, we injected AAV9.CamKII.HI.eGFP-Cre.WPRE.SV40 in ReaChR or Ai32 mice in the right ALM (anterior 2.5 mm from bregma, lateral 1.5 mm, depth 0.5 and 0.8 mm, 100 nl at each depth), left Thal_ALM_ (posterior 1.5 mm, lateral 0.8 mm, depth 4.1 mm, 150 nl) or left S1/S2. To target a region spanning vS1 and S2, the left hemisphere was tilted down by 5° from the horizontal plane and the injection pipettes entered the brain vertically at posterior 1.5 mm and lateral 4 mm from bregma. Virus was injected at depths 0.8, 1.2 and 2 mm (100 nl at each depth). To characterize long-range input connectivity in vM1 (Extended Data Fig. 6), we injected AAV9.CamKII.HI.eGFP-Cre.WPRE.SV40 (Penn Vector Core, University of Pennsylvania) in Ai32 mice in vS1 (posterior 1.0 mm from bregma, lateral 3.1 mm, depth 0.5 and 0.8 mm, 100 nl at each depth) or M2 (anterior 2.7 mm, lateral 0.9 mm, depth 0.5 and 0.8 mm, 100 nl at each depth).

To quantify the fraction of anterogradely labeled neurons in ALM due to potential tropism of the Cre viruses (Extended Data Fig. 6e), we collected 30-µm free-floating coronal sections around ALM (three mice each for S1/S2, cALM and Thal_ALM_ injections). Sections were stained with NeuN. Regions covering ALM layers 2/3 and 5 were imaged with an LSM710 (Zeiss) and processed with ImageJ. Cell counting was performed manually (Extended Data Fig. 6e).

### Photostimulation

#### ChR2-tagging and ChR2-assisted circuit mapping

Photostimulation and electrophysiology recordings were performed in the left ALM to photostimulate ChR2- or ReaChR-expressing axons from left S1/S2, right ALM or left Thal_ALM_. Light from a 473-nm (UltraLasers, MBL-FN-473-300 mW) or 593-nm laser (UltraLasers, MGL-N-593.5-200 mW) was controlled by an acousto-optical modulator (Quanta Tech, MTS110-A3-VIS), and focused onto the brain surface through a craniotomy (beam diameter: 400 µm at 4σ). For whole-cell recordings, photostimulation consisted of four powers (1, 5, 10, 20 mW) and four pulse conditions (1, 3, 5, 10 pulses; 2-ms pulses at 5-ms interval). For silicon probe recordings, photostimulation consisted of three powers (10, 20, 30 mW) and three light pulses (1-ms pulses at 200-ms interval). Photostimulation was tested outside of the behavioral task.

#### Photoinhibition of S1/S2, cALM and Thal_ALM_

For photoinhibition of S1/S2 and cALM, we photostimulated GABAergic neurons in VGAT-ChR2-EYFP, PV-ires-cre × Ai32 or PV-ires-cre × ReaChR mice. These methods resulted in similar photoinhibition^44^. Light was delivered through an optic fiber placed on the clear skull implant (S1/S2, bregma posterior 1.5 mm, lateral 4 mm; cALM, anterior 2.5 mm, lateral 1.5 mm). We used 40-Hz photostimulation with a sinusoidal temporal profile. The duration was 1.3 s including a linear ramp during laser offset (100 or 200 ms). The average power was 4 mW. In a subset of S1/S2 photoinhibition, 8 mW was used. At this power, the photoinhibition silenced activity in a cortical area of 2-mm radius (at half-max) through all cortical layers^44^. For photoinhibition of Thal_ALM_, we photostimulated the thalamic reticular nucleus axons in VGAT-ChR2-EYFP mice^25^. Photostimulation was through an optic fiber (Thorlabs, Part no. CFMLC22L05) implanted above ventral-medial nucleus/VAL (bregma posterior 1.5 mm, lateral 0.8 mm, depth 4.1 mm). The average power was 3 mW measured at the tip of the optic fiber. In these experiments, photostimulation occurred during either the sample or delay epoch randomly in 25% of trials.

We also directly photoinhibited Thal_ALM_ using soma-targeted GtACR1 (ref. ^46^). AAV9.CamKII.HI.eGFP-Cre.WPRE.SV40 (University of Pennsylvania Vector Core) was injected in ventral-medial nucleus/VAL (posterior 1.5 mm from bregma, lateral 0.8 mm, depth 4.1 mm, 120 nl) of R26-LNL-GtACR1-Fred-Kv2.1 mice^44^. In these experiments, photostimulation occurred during the sample, delay or response epoch randomly in 25% of trials. The average power was 1–3 mW.

To prevent mice from distinguishing photostimulation trials from control trials using visual cues, a masking flash (1-ms pulses at 10 Hz) was delivered using 470-nm or 590.6-nm light-emitting diodes (Luxeon Star) near the eyes of the mice. The masking flash began as the pole started to move and continued through the end of the epoch in which photostimulation could occur.

### Electrophysiology

#### Silicon probe recordings

A craniotomy (diameter < 1 mm) was made over the left ALM. A silicon probe was acutely inserted 0.9–1.11 mm below the brain surface. To minimize brain movement, a drop of silicone gel (3-4680, Dow Corning) was applied over the craniotomy after the electrode was in the tissue. The tissue was allowed to settle for 15 min before the recording started. Extracellular spikes were recorded using 64-channel Cambridge NeuroTech silicon probes (H2 acute probe, 25-µm spacing, 2 shanks). The voltage signals were amplified and digitized on an Intan RHD2164 64-Channel Amplifier Board (Intan Technology) at 16 bits, recorded on an Intan RHD2000-Series Amplifier Evaluation System (sampling at 20,000 Hz) and stored for offline analysis. Two to eight recordings were made from each craniotomy. DiI was applied to the tip of the silicon probe in the last session to label the recording tracks.

#### Whole-cell recording

A craniotomy (diameter, 100–200 µm) was made in vM1 or ALM. Recordings were obtained using a glass pipette (tip resistance, 7–11 MΩ) and MultiClamp 700B amplifier (Molecular Devices). The signal was sampled at 20 kHz using Wavesurfer (http://wavesurfer.janelia.org/). Membrane potential was not corrected for liquid junction potential. The intracellular solution contained (in mM): 128 potassium gluconate, 4 MgCl_2_, 10 HEPES, 1 EGTA, 4 Na_2_ATP, 0.4 Na_2_GTP, 10 sodium phosphocreatine (pH 7.23; 283 mOsm). Aliquoted ATP/GTP was added to the internal solution on the day of recording. Positive pressure (200 mBar) was applied before insertion to reduce pipette tip contamination. Then, 1.5% agar (Sigma, A1296) in artificial cerebrospinal fluid (aCSF) was applied over the craniotomy after the pipette tip reached pia surface. Recording depth was based on manipulator reading. The series resistance was monitored through a current pulse (100 ms, −0.2 nA) injection. Only neurons with GΩ-seal were included for analysis. Once a GΩ-seal was achieved, an increasing negative pressure was applied slowly until break-in was established. A family of step currents (500 ms or 750 ms, in 40-pA steps) were injected in current-clamp mode (Extended Data Fig. 7). Each craniotomy was used for 1–2 recording sessions.

In calibration recordings, 1 mM TTX (Tocris Bioscience) and 100 µM 4-AP (Acros Organics) were applied topically over the recording craniotomy to verify synaptic connection with long-range input axons. TTX was a sodium channel blocker that prevented local action potential transmission and excitation in unconnected neurons (Fig. 5e,f and Extended Data Fig. 6a,b). Unlike ChR2-assisted circuit mapping in vitro, we found that 4-AP was not required to elicit light-induced EPSPs in connected neurons during application of TTX. To confirm the EPSPs arose from glutamatergic transmission, 20 mM NBQX (Tocris Bioscience) and 30 mM AP5 (Tocris Bioscience) were applied topically to block glutamate receptors. This abolished the EPSPs (Extended Data Fig. 6b).

### Behavioral data analysis

We separately computed performance for ‘lick right’ and ‘lick left’ trials as the fraction of correct choices, excluding lick early trials and ignore trials (Fig. 4b). Significance of the performance change in each photostimulation condition was determined using a nested bootstrap to account for variability across mice, sessions and trials^26^. We tested against the null hypothesis that the performance change caused by photostimulation was due to normal behavioral variability. In each round of bootstrap, we replaced the original behavioral dataset with a resampled dataset in which we resampled with replacement from: (1) mice, (2) sessions performed by each mouse, (3) the trials within each session. We then computed the performance change on the resampled dataset. Repeating this procedure 10,000 times produced a distribution of performance changes that reflected the behavioral variability. The *P* value of the observed performance change was computed as the fraction of times the bootstrap produced an inconsistent performance change (for example, if a performance decrease was observed during photostimulation, the *P* value was the fraction of times a performance increase was observed during bootstrap).

### Electrophysiology data analysis

#### Silicon probe recording preprocessing

The extracellular recording traces were band-pass filtered (300–6 kHz). Events that exceeded an amplitude threshold (4 s.d. of the background) were subjected to manual spike sorting to extract single units^26^. The primary dataset consisted of 9,626 single units, 73 mice, 347 sessions. The second dataset consisted of 10,420 neurons, 29 mice, 110 sessions.

Spike width was the trough-to-peak interval in the mean spike waveform. Units with spike width < 0.35 ms were defined as fast-spiking neurons (1,045 of 20,046) and units with spike widths > 0.45 ms as putative pyramidal neurons (18,266 of 20,046). Units with intermediate values (0.35–0.45 ms, 735 of 20,046) were excluded from analyses. This classification was previously verified by optogenetic tagging of GABAergic neurons^26,44^. Unless stated otherwise, we concentrated our analyses on the putative pyramidal neurons.

#### *t*-SNE and clustering analysis of individual neuron response profiles

We computed each neuron’s average PSTHs for ‘lick left’ and ‘lick right’ trials (correct trials only) and concatenated the PSTHs. Each PSTH was baseline-subtracted and magnitude-normalized by dividing by the norm of the PSTH. We excluded neurons that did not exhibit consistent PSTHs. Specifically, we split each neuron’s trial data in half and computed PSTHs twice using the split data. We then computed Pearson’s correlation between the PSTHs. Neurons with correlation coefficient less than 0.5 were excluded (3,970 of 20,046). In whole-cell recordings, some neurons did not produce enough spikes to calculate PSTH. However, we found that the PSTH calculated from trial-averaged membrane potential (*V*_m_) closely matched the spiking activity PSTH^27^. We therefore used PSTHs calculated from *V*_m_ for the whole-cell data. *V*_m_ was downsampled in time to match the PSTHs from spiking activity.

The input data for the *t*-SNE were an *n* × *t* matrix, where the rows contain the PSTHs of individual neurons. We tested a range of parameters for *t*-SNE, including perplexity (30 to 1,600), distance metrics (correlation, cosine or Euclidean distance) and the number of principal components (20–100). Only the perplexity affected the embedding outcome, but the results were similar for perplexity 30–100. We therefore used perplexity of 50, 50 principal components and cosine distance for the embedding. We computed *t*-SNE ten times and picked the outcome with the lowest Kullback–Leibler divergence. We performed *t*-SNE separately on the primary and second datasets.

#### ePAIRS test for clustering of response profiles

To test if ALM neurons exhibited clusters of prototypical response profiles or a uniform continuum of response profiles, we used the projection angle index of response similarity (PAIRS) test first presented by ref. ^9^. We used a modified version of the PAIRS test presented by ref. ^12^ which accounted for the variance structure of the data, that is, ePAIRS test.

The input data were an *n* × *t* population response matrix, where the rows contain the PSTHs of individual neurons (‘lick left’ and ‘lick right’ trials concatenated). The PSTHs were baseline-subtracted and magnitude-normalized. We used PCA to reduce the dimensionality from *n* to 26, capturing 98% of the activity variance over time. We then examined the loadings matrix (*n* × 26), which represented the weights of individual neurons for the 26 principal components. Each neuron’s response profile over time was thus represented by a 26-element vector. For each neuron, we computed the average vector angle between the neuron and its *k* nearest neighbors. Across the population of neurons, we obtained a distribution of average angles. The median of this distribution should be small if neurons exhibited similar response profiles.

For comparison, we generated null distributions that exhibited no clustering. We drew *n* samples from a 26-dimensional multivariate Gaussian distribution using the MATLAB function *mvnrnd()*. As in the ePAIRS test presented by ref. ^12^, we drew samples from a multivariate distribution with zero mean and the variance along each dimension was matched to the neural data. We then computed the average vector angles of nearest neighbors for this simulated dataset. This yielded null distributions for neuronal populations with a uniform continuum of PSTH shapes. To statistically compare the null distributions and the empirical distribution, we simulated the null distribution 10,000 times and calculated the fraction of times the median of the empirical distribution exceeded the median of the null distribution. This corresponded to a *P* value.

The average vector angle depended on the number of dimensions considered. Here we used 26 principal components, but we also tried as few as eight principal components (the same as in ref. ^9^) and reached the same statistical outcome. The average vector angle also depended on the parameter *k*. We tested a range of *k* (1–10) and arrived at the same statistical outcome. Following ref. ^12^, we used simulated data to validate the ePAIRS test. We drew samples from either a single multivariate normal distribution (that is, no clustering) or from multiple multivariate normal distributions (that is, multiple clusters). We found that the ePAIRS test appropriately captured only cases where samples originated from multiple distributions.

#### Clustering of response profiles

Density peak clustering^63^ was performed in the two-dimensional *t*-SNE representation. Clustering using the top principal components also produced similar results, but clustering in the *t*-SNE gave slightly more consistent PSTHs within clusters. Density peak clustering required manual selection of clusters based on local density. We evaluated the robustness of cluster number across a range of population size. Subpopulations were created by subsampling neurons in the dataset and clusters were selected blind to the population size. The number of clusters saturated at ~100 (Extended Data Fig. 1e). To correct for over-clustering, we manually examined the PSTHs of each cluster and combined a small number of clusters (<10%) with very similar PSTHs. The primary dataset yielded 94 clusters. *t*-SNE and clustering were performed independently on the second dataset, resulting in 86 clusters.

To examine the consistency of response profiles between the primary and second datasets (Fig. 1f,g and Extended Data Fig. 1f,g), we matched clusters across the two datasets. Because the second dataset has a longer delay epoch (1.7 s), we downsampled the PSTHs in the delay epoch using the MATLAB function *resample()*. For each cluster from the second dataset, we computed Pearson’s correlation for its PSTHs (‘lick left’ and ‘lick right’ trials concatenated) with all the clusters from the primary dataset. The clusters were matched based on the highest correlation coefficient (Extended Data Fig. 1f). In some cases, the cluster with the highest correlation coefficient had already been matched to another cluster. The matched clusters were then defined as the next best match based on Pearson’s correlation coefficient and visual inspection of the PSTHs. We did not exhaustively match all clusters of the two datasets. Rather, we focused on a subset of the clusters from the second dataset (48 of 86) that could be easily matched to a cluster from the primary dataset.

To visualize the response profiles of all clusters (Fig. 1f and Extended Data Fig. 1h), the clusters were first sorted into three groups: lick right preferring, lick left preferring and nonselective. Within each group, clusters were further sorted by activity onset time. For the nonselective clusters, clusters were further subdivided into excitatory and suppressive responses before sorting by activity onset time.

#### Cluster reproducibility

To evaluate the robustness of clusters from density peak clustering, we also performed Louvain–Jaccard clustering on the primary dataset. We calculated the matrix for co-clustering of every pair of neurons in each method (Extended Data Fig. 1c). We sorted the neurons based on co-clustering in density peak clustering. The block structure along the diagonal of the cell–cell co-clustering matrix was preserved in Louvain–Jaccard clustering, which indicates that if two cells belonged to the same cluster in density peak clustering, then their co-clustering probability was high for Louvain–Jaccard clustering.

To define reproducible clusters, for each cluster in density peak clustering, we found its matching cluster in Louvain–Jaccard clustering. A matching cluster must have >50% of its units also present in the original cluster. By this criterion, 70 of 94 clusters from density peak clustering could be matched to a cluster in Louvain–Jaccard clustering. For the matched clusters, 59 of 70 clusters defined by density peak clustering had >50% of their units captured by their matching clusters defined by Louvain–Jaccard clustering. We considered these clusters to be reproducible. The irreproducible clusters tend to be small in size (Extended Data Fig. 1d), representing 25.8% of the neurons in the dataset.

#### Noise correlation

We calculated noise correlation between all simultaneously recorded neuron pairs with three constraints (Fig. 1h and Extended Data Fig. 1i). First, neuron pairs must be >100 µm apart. This avoided contamination of spikes from spike sorting. Second, each neuron must be recorded for >10 trials for each trial type. Third, each neuron was only used once in neuron pairs. This avoided using the same neuron in multiple neuron pairs, making neuron pairs nonindependent from each other in statistical tests. In total, we obtained 1,060 within-cluster pairs out of 2,658 possible neuron pairs, and 1,598 across-cluster pairs instead of 107,136 possible pairs. Noise correlation was calculated separately for ‘lick right’ and ‘lick left’ trials and separately during baseline, sample, delay and response epochs. The baseline epoch was 500 ms before the sample epoch. Only correct trials were used. For each trial, we calculated spike counts within 100-ms windows. For each time window, we subtracted the mean spike count calculated from all trials of the same trial type. Noise correlation was Pearson’s correlation of the mean-subtracted spike counts across trials and time windows.

#### Decoding

Decoding was performed independently at each time point (200-ms windows in 50-ms steps). Decoding was performed using a linear support vector machine on pseudo-populations that combined neurons from different recordings. We concatenated the single-trial spike counts of individual neurons to generate population response vectors. Because neurons were not recorded simultaneously, trials from different neurons were randomly matched. This approach ignored any trial-to-trial correlation between neurons. Response vectors for testing were built using trials that were not used for training. Decoding was repeated 20 times using population responses generated from different combinations of single neuron trial data. Standard errors of mean performance were calculated as the standard deviations of performances across different runs. We used pseudo-populations because most recording sessions did not yield many neurons simultaneously recorded for enough error trials.

To decode trial type, we matched the number of correct and error ‘lick left’ and ‘lick right’ trials. Trials could be classified by stimulus, choice or outcome while holding the other two variables constant^29,31,32^. To decode trial epochs, correct ‘lick left’ and ‘lick right’ trials were combined. Population response vectors were subjected to four-way classification (baseline, sample, delay or response epochs). To decode reaction time, correct ‘lick left’ and ‘lick right’ trials were combined and sorted by reaction time (interval between ‘go’ cue onset and the first lick). The top and bottom one-third of the sorted trials were used for binary classification. To decode ignore trials, all ‘lick left’ and ‘lick right’ trials were combined and classified by whether mice generated a licking response within a 1.5-s time window following the ‘go’ cue.

#### Population selectivity vectors

Across *n* neurons, we concatenated the trial-averaged responses within specific time windows into *n* × 1 response vectors that described the population response for each trial type: correct ‘lick right’ trials, $\overline {{{{\boldsymbol{CR}}}}}$; correct ‘lick left’ trials, $\overline {{{{\boldsymbol{CL}}}}}$; error ‘lick right’ trials, $\overline {{{{\boldsymbol{ER}}}}}$; error ‘lick left’ trials, $\overline {{{{\boldsymbol{EL}}}}}$. The population selectivity vectors were calculated as:

$$\begin{array}{l}{\mathrm{Stimulus}}\,{\mathrm{selectivity}}\,{\mathrm{vector}} = \frac{{\left( {\overline {{{{\boldsymbol{CR}}}}} - \overline {{{{\boldsymbol{EL}}}}} } \right) + \left( {\overline {{{{\boldsymbol{ER}}}}} - \overline {{{{\boldsymbol{CL}}}}} } \right)}}{2}\\ {\mathrm{Choice}}\,{\mathrm{selectivity}}\,{\mathrm{vector}} = \frac{{\left( {\overline {{{{\boldsymbol{CR}}}}} - \overline {{{{\boldsymbol{ER}}}}} } \right) + \left( {\overline {{{{\boldsymbol{EL}}}}} - \overline {{{{\boldsymbol{CL}}}}} } \right)}}{2}\\ {\mathrm{Outcome}}\,{\mathrm{selectivity}}\,{\mathrm{vector}} = \frac{{\left( {\overline {{{{\boldsymbol{CR}}}}} - \overline {{{{\boldsymbol{ER}}}}} } \right) + \left( {\overline {{{{\boldsymbol{CL}}}}} - \overline {{{{\boldsymbol{EL}}}}} } \right)}}{2}\end{array}$$

Stimulus selectivity represents the response in posterior object location trials (CR, ER) relative to anterior trials (CL, EL). Choice selectivity represents the response when mice licked right (CR, EL) relative to licking left (CL, ER). Outcome selectivity represents the response in correct trials (CR, CL) relative to error trials (ER, EL).

Selectivity vectors were calculated in analysis windows of 100 ms at different time points (1-ms steps). We quantified the similarity of selectivity vectors across time using Pearson’s correlation (Fig. 2b, off diagonal). Within each analysis window, we calculated Pearson’s correlation between the selectivity vectors calculated from split-half trials (Fig. 2b, diagonal).

#### Activity modes

Across *n* neurons, we defined a set of orthogonal directions in activity space (**Mode**, *n* × 1 vectors) that captured components of population activity (Fig. 2). We defined the activity modes using a portion of the trials. Separate trials were used for activity projections. At each time point, we calculated the trial-averaged population response vectors (***r***, *n* × 1) for specific trial types. Activity projections were calculated as **Mode**^*T*^***r***. To obtain standard errors, we bootstrapped the neurons in the dataset. Standard error was the standard deviation of the activity projections calculated on the resampled datasets.

We calculated stimulus, choice and outcome modes from selectivity vectors (see Population selectivity vectors). Stimulus selectivity vectors were similar during the sample and early delay epochs (Fig. 2b). We averaged the stimulus selectivity vectors in the sample epoch to obtain the stimulus mode, ${{{{{\mathbf{Mode}}}}}}_{{\mathrm{stimulus}}} = \frac{{\left( {\overline {{{{\boldsymbol{CR}}}}} - \overline {{{{\boldsymbol{EL}}}}} } \right) + \left( {\overline {{{{\boldsymbol{ER}}}}} - \overline {{{{\boldsymbol{CL}}}}} } \right)}}{2}$. Choice selectivity vectors developed during the late sample epoch and were stable during the delay epoch (Fig. 2b). We averaged the choice selectivity vectors in the delay epoch to obtain the choice mode, ${{{{\mathbf{{Mode}}}}}}_{{\mathrm{choice}}} = \frac{{\left( {\overline {{{{\boldsymbol{CR}}}}} - \overline {{{{\boldsymbol{ER}}}}} } \right) + \left( {\overline {{{{\boldsymbol{EL}}}}} - \overline {{{{\boldsymbol{CL}}}}} } \right)}}{2}$. We defined the action mode based on choice selectivity during movement initiation (0.1 s < *t* < 0.3 s after the ‘go’ cue), ${{{{{\mathbf{Mode}}}}}}_{{\mathrm{action}}} = \overline {{{{\boldsymbol{CR}}}}} - \overline {{{{\boldsymbol{CL}}}}}$. We defined the outcome mode by averaging the outcome selectivity vectors during the response epoch (0 s < *t* < 1.3 s after the ‘go’ cue), ${{{{{\mathbf{Mode}}}}}}_{{\mathrm{outcome}}} = \frac{{\left( {\overline {{{{\boldsymbol{CR}}}}} - \overline {{{{\boldsymbol{ER}}}}} } \right) + \left( {\overline {{{{\boldsymbol{CL}}}}} - \overline {{{{\boldsymbol{EL}}}}} } \right)}}{2}$.

We additionally defined two non-trial-type-selective activity modes previously shown to play roles in decision-making and motor response^30,35^. We defined a ramping mode as ${{{{{\mathbf{Mode}}}}}}_{{\mathrm{ramping}}} = {{{\bar{\boldsymbol r}}}}_{delay} - {{{\bar{\boldsymbol r}}}}_{pre\,sample}$, where ${{{\bar{\boldsymbol r}}}}_{pre\,sample}$ represents the population response vector 500 ms before the sample epoch and ${{{\bar{\boldsymbol r}}}}_{delay}$ represents the population response vector during the last 500 ms of the delay epoch. We defined a go mode that captured the phasic activity after the ‘go’ cue^35^, ${{{{{\mathbf{Mode}}}}}}_{{\mathrm{go}}} = {{{\bar{\boldsymbol r}}}}_{after\,go\,cue} - {{{\bar{\boldsymbol r}}}}_{before\,go\,cue}$, where ${{{\bar{\boldsymbol r}}}}_{before\,go\,cue}$ and ${{{\bar{\boldsymbol r}}}}_{after\,go\,cue}$ represent the population response vectors 100 ms before and after the ‘go’ cue. The ramping and go modes were calculated using the combined responses from correct ‘lick left’ and ‘lick right’ trials.

Finally, we calculated an activity mode that captured most of the remaining activity variance. We calculated eigenvectors of the population response using singular value decomposition (SVD). The data for the SVD were an *n* × *t* population response matrix containing the baseline-subtracted PSTHs of *n* neurons (‘lick right’ and ‘lick left’ trials concatenated). Consistent with previous analyses of frontal cortex activity^7,41^, the eigenvector carrying the most variance showed non-trial-type-selective modulation during the response epoch, which we defined as the response mode (**Mode**_response_).

The seven activity modes were near orthogonal to each other (Extended Data Fig. 2a). For all analyses in the paper, we further rotated the activity modes using the Gram–Schmidt process to be fully orthogonal to each other. Together, the seven activity modes captured 69% of ALM activity variance, 71% of the stimulus selectivity, 92% of the choice selectivity and 93% of the outcome selectivity (Extended Data Fig. 2b). Activity variance was the root mean square (r.m.s.) of the baseline-subtracted activity over the sample, delay and response epochs. The population stimulus and choice selectivity were the r.m.s. values of the stimulus and choice selectivity over the sample and delay epochs. The population outcome selectivity was the r.m.s. of the outcome selectivity during the response epoch.

Our primary analyses were based on neurons combined from different recordings. We also performed the same analysis on simultaneously recorded populations (Extended Data Fig. 2c). We restricted the analysis to sessions with at least ten neurons recorded simultaneously for at least ten trials of each trial type (33 sessions, 10–57 neurons recorded simultaneously, 24 neurons on average). To average activity projections across multiple sessions, we offset the activity projections of each session by subtracting the global mean of activity projections across all trials and time points. This removed session-to-session fluctuations in mean activity. The offsets were computed using the trials that were used to construct the activity modes. Independent trials were used for activity projections.

#### Activity modes from demixed PCA

Demixed PCA was performed using the dPCA package, https://github.com/machenslab/dPCA (v.1.0.5)^7^ (Extended Data Fig. 2c,d). The input to the dPCA was an *n* × *s* × *d* × *t* × *k* matrix where each entry was the spike rates of individual neurons in individual trials (calculated in 200-ms windows). *n* corresponds to neurons, *s* corresponds to trial types instructed by object location (‘stimulus’, anterior versus posterior), *d* corresponds to lick directions (‘choice’, left versus right), *t* corresponds to time steps (1 ms) and *k* corresponds to individual trials. Neurons were combined from different recordings. For conditions with fewer numbers of trials, the empty entries in the response matrix were filled in with *nan’s*. dPCA was performed using the MATLAB function *dpca()* in the package with the default parameters. We found that ALM population activity reorganized dramatically after the ‘go’ cue (Fig. 2b). We therefore used the *timeSplits* option in the *dpca()* function to split the time periods at the ‘go’ cue for separate marginalization.

#### ePAIRS test for mixed selectivity

We tested for a notion of mixed selectivity where a shared neuronal population encoded random mixtures of the seven activity modes defined above (Fig. 2). Each activity mode corresponds to a weighted sum of individual neuron activities. Each neuron’s weights for the activity modes constituted a seven-dimensional coding vector. We calculated the angles between each neuron’s coding vector and its nearest neighbors. We then tested if the distribution of the nearest-neighbor angles differed from null distributions expected from random distribution of coding vectors. For null distribution, we drew vectors from a seven-dimensional multivariate Gaussian distribution with variance along each dimension matched to the neural data. The procedures for the ePAIRS test were the same as above (see ePAIRS test for clustering of response profiles), but here for the seven-dimensional vectors (Fig. 3c).

#### Joint coding of specific activity modes

We characterized individual neurons’ weights for pairs of activity modes as two-dimensional vectors (Fig. 3d). If a pair of activity modes were randomly mixed across a shared neuronal population, the angles of the coding vectors would be uniformly distributed. If neurons coded individual activity modes, the distribution would exhibit peaks at 0° and 90°. Because weights can take on positive or negative values, we limited the angles to 0° and 90° by taking the absolute value of the weights. Finally, we limited the analysis to the top 20% of the neurons ranked by the length of their coding vectors. If neurons were not selective for either activity mode, their coding vectors would appear uniformly distributed even though the vector lengths were very small. Importantly, this selection did not affect the coding vector distribution because neurons coding both activity modes also exhibited large vector length. Using a more inclusive criterion (for example, the top 50% of the neurons) yielded similar results.

#### Synthetic neuronal population

We generated a synthetic population coding random mixture of activity modes (Fig. 3c,d and Extended Data Fig. 4e–g). We first computed the seven activity modes as described above (see Activity modes). We then found eigenvectors of the population response matrix (*n* × *t*) using SVD. The population response matrix contained the baseline-subtracted PSTHs of *n* neurons (‘lick right’ and ‘lick left’ trials concatenated). We rotated the eigenvectors using the Gram–Schmidt process to be orthogonal to the seven activity modes. Individual neuron PSTHs could be reconstructed from linear combinations of the activity modes and eigenvectors. We constructed synthetic neuron responses using random combinations of the activity modes and eigenvectors (Extended Data Fig. 4e,f). The weights were drawn from a Gaussian distribution with zero mean. The variance of the Gaussian distribution was scaled so each activity mode and eigenvector in the synthetic population carried the same amount of activity variance as in the original population. This procedure thus preserved the activity modes, but randomly redistributed them across the synthetic population. We calculated *t*-SNE of the synthetic responses (Extended Data Fig. 4g). We also recalculated the activity modes using the synthetic responses and obtained the weights of individual neurons (Extended Data Fig. 4g). We carried out the ePAIRS test as described above on the synthetic population (Fig. 3c and Extended Data Fig. 4b).

#### Functional populations

We performed *k*-means clustering on the activity mode weights (*n* × 7 matrix for a population of *n* neurons) to divide neurons into functional populations (Fig. 3e and Extended Data Fig. 5a,b). Only neurons with more than five error trials of each trial type are included for this analysis. For the stimulus, choice and action modes, large positive and negative weights both indicated strong contributions to the activity modes, but with opposite preference for trial types (Fig. 3b). We therefore took the absolute value of the weights before clustering. We tested a range of cluster numbers, and six clusters produced the largest Silhouette score (Euclidean distance).

For each activity mode, we quantified the fraction of its variance carried by each functional population (Extended Data Fig. 5a,b). We calculated activity projection using only neurons from a functional population, that is, by setting the weights of other neurons to zero (Fig. 3a). The variance of the activity projection is divided by the variance of the full population activity projection to calculate the fraction of variance carried.

To classify functional population identify based on *t*-SNE location (Fig. 3f), we used a nearest-neighbor classifier. Each neuron is classified based on the identity of its ten nearest neighbors in the *t*-SNE.

#### Effects of S1/S2, cALM and Thal_ALM_ photoinhibition

To quantify the effect of photoinhibition on ALM neuron spike rates (Fig. 6 and Extended Data Fig. 10), we calculated spike counts within the photoinhibition window and compared them with the control trials in the same time window. Significant spike rate change was tested using two-tailed *t*-test (*P* < 0.01). ‘Lick left’ and ‘lick right’ trials were pooled. Photoinhibition during the sample and delay epochs was pooled.

To quantify the effect of photoinhibition on ALM activity modes (Fig. 7 and Extended Data Fig. 10), we projected activity in control and photostimulation trials on the activity modes. Because S1/S2, cALM and Thal_ALM_ photoinhibition were tested on different sessions, activity modes were computed separately for each condition. Activity modes were calculated using a subset of control trials. Separate trials were used for activity projections. For activity projections, both correct and error trials were used (Figs. 7 and 8), grouped by instructed trial type. We calculated the difference in activity projections between control and photostimulation trials in the photoinhibition window. Because the difference could be positive or negative, we took the absolute value of the difference. For each activity mode, the activity change was standardized by dividing by the standard deviation of the control trial activity projection across time. ‘Lick left’ and ‘lick right’ trials were pooled. Sample and delay epoch photoinhibition were pooled.

We compared the activity change of Thal_ALM_ versus S1/S2 or cALM photoinhibition (Fig. 7d). Significance was determined by bootstrap. In each round of bootstrap, we resampled neurons in the dataset with replacement. Activity change was calculated on the resampled dataset. Repeating this procedure 10,000 times produced a distribution of activity changes that reflected the variability from neuron sampling. A *P* value was computed as the fraction of times the bootstrap produced an inconsistent activity change (for example, if Thal_ALM_ photoinhibition produced a stronger activity change than S1/S2 in the data, the *P* value was the fraction of times S1/S2 produced a stronger activity change during bootstrap).

#### ChR2-tagging and silicon probe recording analysis

To identify putative postsynaptic neurons (Fig. 5b), tagged neurons were defined based on time-locked responses to the photostimulation (10–30 mW). We compared the spike counts in 20-ms windows before and following each light pulse (1 ms). Significant change was tested using two-tailed *t*-test (*P* < 0.01). Among the significantly excited neurons, we quantified the average number of spikes evoked per light pulse relative to baseline spike rate. Latency was the first time bin in which the baseline-subtracted spike rate reached 50% of the peak amplitude. Neurons with latency less than 5 ms and >0.2 spikes evoked per light pulse were deemed putatively connected. The fraction of connected neurons in each functional population was relative to all the tested neurons in that population (Fig. 5g). Standard error was calculated by bootstrapping the entire dataset (regardless of whether neurons were tested for connectivity). Standard error was the standard deviation of the fractions calculated on the resampled dataset.

#### Whole-cell recording analysis

Neurons with resting membrane potential (*V*_m_) below −40 mV were included for analysis. Spikes were clipped off by interpolating the *V*_m_ before (−1 ms) and after (4 ms) each spiking event. Light-evoked EPSP was the baseline-subtracted *V*_m_. Baseline *V*_m_ was averaged in a 10-ms window before photostimulation. Mean EPSP was calculated in a 20-ms window after laser onset. Latency was when EPSP reached 10% of its peak. In calibration recordings, long-range input connections were verified with TTX pharmacology (Fig. 5d–f and Extended Data Fig. 6). At 20 mW, an EPSP latency threshold of 5 ms could reliably distinguish ALM neurons with S1/S2, cALM and Thal_ALM_ input connections (Fig. 5e,f). We thus used this latency threshold to infer connections in recordings during behavior (Fig. 5g). In vM1, the EPSP latency was faster than ALM neurons (Extended Data Fig. 6c). Nevertheless, connected and unconnected neurons could still be differentiated based on latency.

Additional analyses ensured that in vivo ChR2-assisted circuit mapping measured long-range input connectivity. First, injection of AAV Cre viruses in the input brain regions might anterogradely infect ALM neurons to express ChR2 or ReaChR, which could contribute to the light-evoked EPSPs. However, post hoc histological analysis showed that anterogradely labeled neurons were rare in ALM (Extended Data Fig. 6e). One mouse was excluded from our analysis due to a slightly higher fraction of labeled ALM neurons. Second, our in vivo ChR2-assisted circuit mapping replicated input connectivity patterns previously measured in slice experiments. In vM1, we found that vS1 inputs preferentially targeted the superficial layers, whereas M2 inputs preferentially targeted the deep layers (Extended Data Fig. 6d)^20^.

Neurons were characterized by firing patterns to current injections (Extended Data Fig. 7c). A regular spiking neuron was defined by characteristic spike frequency adaptation. A bursting neuron was defined by short inter-spike interval and amplitude decrease in a train of spikes. A high-threshold neuron was defined by a lack of spiking activity upon high-amplitude current injection (500 or 1,000 pA). Neurons were also characterized by EPSP amplitude to a train of light pulses (Extended Data Fig. 7d). Increasing EPSP amplitude defined facilitating synapses. Decreasing amplitude defined depressing synapses. Unconnected neurons showed either inhibitory postsynaptic potentials (IPSPs), indicating di-synaptic inhibition, or delayed EPSPs, indicating indirect connection. Finally, 1-ms alternating depolarizing or hyperpolarizing current (500 pA) was injected to measure the membrane time constant (Extended Data Fig. 7e). The time constant was calculated as the slow component of a double-exponential fit of the average *V*_m_ decay^64^.

#### Definition of cortical layers

For analyses across cortical depth (Figs. 3, 5 and 6), we used layer annotations in the Allen Mouse Brain Common Coordinate Framework (CCFv3). We labeled a subset of recording tracks using DiI (eight penetrations, eight mice). We aligned coronal sections containing the labeled tracks into the CCFv3 using an affine transformation followed by a nonrigid transformation using b-splines^49^. The tracks were reconstructed in CCFv3, which provided the layer annotations across depth (boundaries between layers 1 and 2/3, 110 ± 8.2 µm; layers 2/3 and 5, 378.3 ± 21.7 µm; layers 5 and 6, 771.7 ± 71.9 µm; mean ± s.d. across penetrations). Using these boundaries, layers were determined for all neurons from their recording depths, which were obtained from manipulator reading and electrode spacing on the probe.

### Statistics

The sample sizes were similar to sample sizes used in the field: for behavior, three mice or more per condition. No statistical methods were used to determine sample size. All key results were replicated in multiple mice. Mice were allocated into experimental groups according to their strain. Unless stated otherwise, the investigators were not blinded to allocation during experiments and outcome assessment. Trial types were randomly determined by a computer program. During spike sorting, experimenters cannot tell the trial type, so experimenters were blind to conditions. Statistical comparisons using *t*-tests, bootstrap and other nonparametric tests are described in detail in the sections above.

### Reporting summary

Further information on research design is available in the Nature Research Reporting Summary linked to this article.

## Online content

Any methods, additional references, Nature Research reporting summaries, source data, extended data, supplementary information, acknowledgements, peer review information; details of author contributions and competing interests; and statements of data and code availability are available at 10.1038/s41593-022-01171-w.

## Supplementary information

Reporting Summary

## Data Availability

Data have been deposited on Zenodo and can be accessed at 10.5281/zenodo.6846161.

## Extended data

are available for this paper at 10.1038/s41593-022-01171-w.

## Code availability

Code for data analysis can be accessed at https://github.com/NuoLiLabBCM/YangEtAl2022_code_activity_modes.
