# Supplementary material for: Thalamus-driven functional populations in frontal cortex support decision-making
Source: Nat Neurosci. 2022 Sep 28;25(10):1339–52. doi: 10.1038/s41593-022-01171-w (PMC9534763; doi:10.1038/s41593-022-01171-w)
Supplement: Supplementary file 1 — Reporting Summary [file 41593_2022_1171_MOESM1_ESM.pdf]

nature portfolio

Corresponding author(s): Nuo Li

Last updated by author(s): Jul 17, 2022

Reporting Summary

Nature Portfolio wishes to improve the reproducibility of the work that we publish. This form provides structure for consistency and transparency in reporting. For further information on Nature Portfolio policies, see our Editorial Policies and the Editorial Policy Checklist.

Statistics

For all statistical analyses, confirm that the following items are present in the figure legend, table legend, main text, or Methods section.

n/a	Confirmed	
☐	☒	The exact sample size (*n*) for each experimental group/condition, given as a discrete number and unit of measurement
☐	☒	A statement on whether measurements were taken from distinct samples or whether the same sample was measured repeatedly
☐	☒	The statistical test(s) used AND whether they are one- or two-sided<br>*Only common tests should be described solely by name; describe more complex techniques in the Methods section.*
☒	☐	A description of all covariates tested
☐	☒	A description of any assumptions or corrections, such as tests of normality and adjustment for multiple comparisons
☐	☒	A full description of the statistical parameters including central tendency (e.g. means) or other basic estimates (e.g. regression coefficient) AND variation (e.g. standard deviation) or associated estimates of uncertainty (e.g. confidence intervals)
☐	☒	For null hypothesis testing, the test statistic (e.g. *F*, *t*, *r*) with confidence intervals, effect sizes, degrees of freedom and *P* value noted<br>*Give P values as exact values whenever suitable.*
☒	☐	For Bayesian analysis, information on the choice of priors and Markov chain Monte Carlo settings
☒	☐	For hierarchical and complex designs, identification of the appropriate level for tests and full reporting of outcomes
☐	☒	Estimates of effect sizes (e.g. Cohen's *d*, Pearson's *r*), indicating how they were calculated

Our web collection on statistics for biologists contains articles on many of the points above.

Software and code

Policy information about availability of computer code

Data collection	Behavioral data was acquired using Bpod <https://www.sanworks.io> and wavesurfer (v 0.787) <https://www.janelia.org/open-science/wavesurf>. Electrophysiological data was acquired using Intan RHD2000-Series Amplifier Evaluation System <https://intantech.com/>
Data analysis	Custom codes written in Matlab 2018b can be accessed at Github <https://github.com/NuoLiLabBCM/YangEtAl2022_code_activity_modes>. PAIRS test was described in Raposo et al. 2014. ePAIRS was described in Hirokawa et al. 2019. demixed PCA, Kobak et al 2016 <https://github.com/machenslab/dPCA > (version 1.0.5). Histology images were processed with ImageJ (version 1.51).

For manuscripts utilizing custom algorithms or software that are central to the research but not yet described in published literature, software must be made available to editors and reviewers. We strongly encourage code deposition in a community repository (e.g. GitHub). See the Nature Portfolio guidelines for submitting code & software for further information.

Data

Policy information about availability of data

All manuscripts must include a data availability statement. This statement should provide the following information, where applicable:

- Accession codes, unique identifiers, or web links for publicly available datasets
- A description of any restrictions on data availability
- For clinical datasets or third party data, please ensure that the statement adheres to our policy

Raw and processed data is available on Zenodo <http://dx.doi.org/10.5281/zenodo.6846161>.

Human research participants

Policy information about studies involving human research participants and Sex and Gender in Research.

Reporting on sex and gender	*Use the terms sex (biological attribute) and gender (shaped by social and cultural circumstances) carefully in order to avoid confusing both terms. Indicate if findings apply to only one sex or gender; describe whether sex and gender were considered in study design whether sex and/or gender was determined based on self-reporting or assigned and methods used. Provide in the source data disaggregated sex and gender data where this information has been collected, and consent has been obtained for sharing of individual-level data; provide overall numbers in this Reporting Summary. Please state if this information has not been collected. Report sex- and gender-based analyses where performed, justify reasons for lack of sex- and gender-based analysis.*
Population characteristics	*Describe the covariate-relevant population characteristics of the human research participants (e.g. age, genotypic information, past and current diagnosis and treatment categories). If you filled out the behavioural & social sciences study design questions and have nothing to add here, write "See above."*
Recruitment	*Describe how participants were recruited. Outline any potential self-selection bias or other biases that may be present and how these are likely to impact results.*
Ethics oversight	*Identify the organization(s) that approved the study protocol.*

Note that full information on the approval of the study protocol must also be provided in the manuscript.

Field-specific reporting

Please select the one below that is the best fit for your research. If you are not sure, read the appropriate sections before making your selection.

☒ Life sciences ☐ Behavioural & social sciences ☐ Ecological, evolutionary & environmental sciences

For a reference copy of the document with all sections, see nature.com/documents/nr-reporting-summary-flat.pdf

Life sciences study design

All studies must disclose on these points even when the disclosure is negative.

Sample size	No statistical methods were used to pre-determine the animal number in our study but our sample sizes are similar to those reported in previous publications. (Gao et al. 2018; Chen, Kang et al. 2021). All key results were replicated in multiple subjects.
Data exclusions	For t-SNE analysis, neurons with inconsistent PSTHs were excluded (3970/20046). In electrophysiological experiments, 735 out of 20046 neurons were excluded from analyses due to their uncertain cell types. This classification has been verified by optogenetic tagging of GABAergic neurons (Guo et al. 2014). For whole-cell recording, one animal was excluded because a higher fraction of anterogradely labeled ALM neurons was found during post hoc examination. These neurons might contaminate the direct long-range projection from brain regions of interest. These exclusions are detailed in the Methods.
Replication	All behavior and recording results were replicated in multiple animals per group (n>=3). All of the data presented in the manuscript as well as histology (Figs 4a and 8a) were successfully replicated in multiple animals (n>=3).
Randomization	Animals of both sexes were randomly assigned to experimental groups. Trial types were randomly determined by a computer program.
Blinding	Analysis of neural and behavior data was conducted regardless of the identity of the animal from which the data was collected. During experiments, trial types were randomly determined by a computer program. During spike sorting, experimenters were blind to the trial type and conditions. Experimenters were not blinded to group allocation for neural and behavioral data analyses. Experiments using optogenetic perturbations to manipulate activity during electrophysiology and behavior require experimenters be aware of mice strain information to ensure expression of opsins. All of the experiments include control conditions within the same mouse (e.g. photostimulation across different behavioral epochs; neurons responsive to photostimulation vs. those do not). Experimenters were blind to conditions during the experiment.

Reporting for specific materials, systems and methods

We require information from authors about some types of materials, experimental systems and methods used in many studies. Here, indicate whether each material, system or method listed is relevant to your study. If you are not sure if a list item applies to your research, read the appropriate section before selecting a response.

Materials & experimental systems

n/a	Involved in the study
☒	☐ Antibodies
☒	☐ Eukaryotic cell lines
☒	☐ Palaeontology and archaeology
☐	☒ Animals and other organisms
☒	☐ Clinical data
☒	☐ Dual use research of concern

Methods

n/a	Involved in the study
☒	☐ ChIP-seq
☒	☐ Flow cytometry
☒	☐ MRI-based neuroimaging

Animals and other research organisms

Policy information about studies involving animals; ARRIVE guidelines recommended for reporting animal research, and Sex and Gender in Research

Laboratory animals	Both male and female transgenic mice were used, including VGAT-ChR2-EYFP, Ai32, Rosa26-LSL-ReaChR-mCitrine, PV-ires-cre crossed with Rosa26-LSL-ReaChR-mCitrine, PV-ires-cre crossed with Ai32, GtACR1 and wildtype mice. Mice were aged 2-5 months old when surgery was performed. Mice were housed at a constant temperature (22±1 °C) and humidity (30-55%)under a 12:12 reverse light:dark cycle and tested during the dark phase.
Wild animals	This study did not involve wild animals.
Reporting on sex	Both male and female mice were used.
Field-collected samples	This study did not contain samples collected from the field.
Ethics oversight	The animal care and surgery procedures were in accordance with the protocols approved by the Institutional Animal Care and Use Committees at Baylor College of Medicine.

Note that full information on the approval of the study protocol must also be provided in the manuscript.
